# Tsallis Extended Thermodynamics Applied to *2-d* Turbulence: Lévy Statistics and *q*-Fractional Generalized Kraichnanian Energy and Enstrophy Spectra

**DOI:** 10.3390/e20020109

**Published:** 2018-02-07

**Authors:** Peter W. Egolf, Kolumban Hutter

**Affiliations:** 1 Thermal Sciences and Engineering Institute, University of Applied Sciences of Western Switzerland, CH-1401 Yverdon-les-Bains, Switzerland; 2 % Laboratory of Hydraulics, Hydrology and Glaciology, Swiss Federal Institute of Technology, ETH, Hönggerberg HIA 58D, CH 8093 Zurich, Switzerland

**Keywords:** extended thermodynamics, Tsallis entropy, escort probability, fractional calculus, *2-d* turbulence, spectra of Kraichnan, Kolmogorov-Oboukov spectrum, Lévy statistics, intermittency

## Abstract

The extended thermodynamics of Tsallis is reviewed in detail and applied to turbulence. It is based on a generalization of the exponential and logarithmic functions with a parameter *q*. By applying this nonequilibrium thermodynamics, the Boltzmann-Gibbs thermodynamic approach of Kraichnan to *2-d* turbulence is generalized. This physical modeling implies fractional calculus methods, obeying anomalous diffusion, described by Lévy statistics with *q* < 5/3 (sub diffusion), *q* = 5/3 (normal or Brownian diffusion) and *q* > 5/3 (super diffusion). The generalized energy spectrum of Kraichnan, occurring at small wave numbers *k*, now reveals the more general and precise result *k^−q^*. This corresponds well for *q* = 5/3 with the Kolmogorov-Oboukov energy spectrum and for *q* > 5/3 to turbulence with intermittency. The enstrophy spectrum, occurring at large wave numbers *k*, leads to a *k^−^*^3*q*^ power law, suggesting that large wave-number eddies are in thermodynamic equilibrium, which is characterized by *q* = 1, finally resulting in Kraichnan’s correct *k^−^*^3^ enstrophy spectrum. The theory reveals in a natural manner a generalized temperature of turbulence, which in the non-equilibrium energy transfer domain decreases with wave number and shows an energy equipartition law with a constant generalized temperature in the equilibrium enstrophy transfer domain. The article contains numerous new results; some are stated in form of eight new (proven) propositions.

## 1. Introduction

There are primarily two limiting cases to describe turbulent flows, a microscopic and a macroscopic description. The first is based on the entire ensemble of atoms and molecules in the flow field. The characteristic amount of a considered entity, respectively the number of degrees of freedom of such a large-scale system, e.g., an atmospheric turbulent flow, is of the order of the value of the Avogadro number (6.022 × 10^23^), which is very large. In 1996 Castaign [1] pointed out that controlling each of these single degrees is impossible and a futile program. The related problem is so large that it can at best be handled by molecular dynamics and supercomputing. On the macroscopic side, it is the description of fluid behavior by the Navier-Stokes Equations (NSE), which reduce the problem to only four macroscopic variables (however, be aware that these variables are functions of space and time), namely three velocity components and the pressure. Naively, one could now assume that by this enormous reduction of variables, which is roughly a factor 10^23^, the problem has been sufficiently simplified, so that it could be easily solved. However, the nonlinear terms in the NSEs lead to another quasi unsolvable problem, which has amply been discussed in the literature (see e.g., [2,3]). Applying Kolmogorov’s microscales, it is easy to show that the number of grid points *N* demanded to solve the NSEs by Direct Numerical Simulation (DNS) is *N* ∝ Re^9/4^ (see e.g., [3]). This leads to an enormous demand of Central Processing Unit (CPU) time to calculate a turbulent flow field of high Reynolds number.

That the two most usual physical concepts show such a high resistance to a solution, makes it attractive to search for alternative mesoscopic or macroscopic models. Such models are e.g., semi-microscopic models, such as the Langevin Equation (LE) and the Fokker-Planck Equation (FPE). Furthermore, also thermodynamic models reduce the degrees of freedom enormously. Because turbulent flows are generically irreversible and non-equilibrium processes, we will further concentrate our efforts on non-equilibrium thermodynamic concepts. Returning to the considerations of Castaign [1], we cite his conclusion that a thermodynamic approach could be advantageous, where only a few important parameters allow a complete description of the large fluid dynamic system. 

We have seen that a successful method is to start to model turbulence by considering the mechanism of Brownian motion. Langevin [4] originally designed his first version of the Langevin Equation (LE) in 1908 (see also [5]) to describe Brownian motion (see [6,7,8]). This equation is a stochastic differential equation that describes only a subset of the entire degrees of freedom. The remaining variables are typically collective and macroscopic variables [9,10]. They change only slowly compared to the faster microscopic variables, which are compacted in a stochastic force term of the Langevin differential equation. With knowledge in this article, it can be foreseen that an analogous version of a Fractional LE (FLE) (see [11,12,13,14]), describing anomalous diffusion processes, could play an essential role in future descriptions of turbulent flows. 

The Fokker-Planck Equation (FPE) (see [15,16]) shows a similar bundling of a high number of degrees of freedom in a time dependent probability distribution of the stochastic variables. In 1931 Kolmogorov independently derived this equation [17]. Applied to particle distributions the equation is called Smoluchovski equation (see e.g., [18]), and if diffusion is missing, in statistical physics, it is called Liouville equation. This partial differential equation describes the temporal evolution of the probability density of the velocities of the particles under the influence of regular forces, but also a random force given by the stochastic properties of microscopic variables e.g., also obeying Brownian movements. Research on a Fractional FPE (FFPE), describing sub diffusion, see e.g., by Barkai [19]).

In old turbulence models, e.g., Prandtl’s mixing length model [20] or mean shear layer model [21], turbulent transport was successfully modeled by enhanced diffusion, named (effective) eddy diffusivity. Today it is slowly becoming clear that there exists a correct link of this concept to anomalous (super) diffusion. However, the Ansatz via Fick’s law in these models is local and linear and, thus, corresponds to a transport of eddies with a single size. It is clear that these models, to obey Lévy statistics, have to be generalized by introducing scaling properties relating to an infinite number of eddy sizes [22]. Egolf and Hutter, by introducing nonlocality and by the application of fractional derivatives to Prandtl’s models [23], proved that the Difference-Quotient Turbulence Model (DQTM) of Egolf, published in 1991 [24], is the natural generalization of two of Prandtl’s turbulence models ([20,21]) to describe turbulent phenomena including intermittency [22,23,24,25,26]. The main development of the DQTM with Lévy statistics borrows its ideas in the microscopic world and applies them to the equally exorbitant high number of eddies in flows of large Reynolds number. However, the main difference is that the largest eddies are of truly macroscopic dimension. Therefore, this special type of approach could be called micro-macroscopic modelling. In this framework, the degrees of freedom are a priori not small, and a reduction of its number is given by the scaling laws. 

Let us return to Castaign’s insight, which is in full agreement with that of Goldenfeld [27]), who in winter 1983–1984 realized that certain non-equilibrium phenomena [like *2-d* turbulence] could be analysed by using renormalization group techniques [28], which at the time was mainly employed in solid state physics for field theoretical approaches and critical phenomena [29], among them also phase transitions [30] to “integrate out” some degrees of freedom. With our conviction that turbulence is a critical phenomenon from today’s point-of-view, this is like closing a circle. Egolf and Hutter discovered a strong analogy between magnetism and turbulence and thereby could develop the mean field theory of turbulence [31]. With this finding they could transform the Curie and the Curie-Weiss laws to the related laws describing turbulence. They call these laws ‘Curie law of turbulence’ and ‘Curie-Weiss law of turbulence’. Calculations originating from the Curie law of turbulence lead to the right response function of turbulence, which they call ‘vorticibility’ and which is a differential form of the turbulence intensity. Based on such ideas, it becomes evident that also thermodynamic models may be successful to reduce the complexity. Especially if the features of nonlinear systems are coupled with non-equilibrium thermodynamic concepts, important new results may be expected. 

It is, however, apt to present an additional side view on activities in which effects described by cooperative dynamics and ordering have been brought into thermodynamic concepts. A mile stone in this area was the phenomenological approach by Prigogine [32]), which gave rise to a new way of studying irreversible thermodynamic systems. Haken (see [9,10]) developed and summarized knowledge on cooperative dynamics in complex physical systems with hierarchical structures (see also [33]). He figured out the slaving principle, where a single mode can reach a superior importance compared to others, and, thus, presents itself as an ideal order parameter of a complex system. This is a procedure, which opens the door for statistical thermodynamic descriptions of nonlinear systems, also showing cooperative phenomena, including phase transitions.

Clothing fluid dynamic systems in a statistical suit started in 1896 by Boltzmann [34], in 1902 by Gibbs [35], and continued in 1949 by Onsager [36] (see also [37,38]). Today mainly two approaches are available: (1) The Euler flow system is developed by a finite number of point vortices (see e.g., [39,40,41,42], and (2) the energy and vorticity are described by a truncated set of (finite) Fourier components ([43,44,45,46,47]). Explicitly, a Hamiltonian can be formulated that contains two constants of the motion [48], which in a *2-d* approach are the kinetic energy and enstrophy and in a *3-d* formulation the kinetic energy and the helicity. This method is the basis for our new theoretical considerations in this article. The loss of information by a wave-number truncation process was tried to be overcome by constructing a Gibbs free energy for the full Euler system (see e.g., [49,50]). Robert [51,52], to obtain the Gibbs potential, goes a similar way as we will also follow in this article (see Section 4), namely, to work with Young measures and the Kullback-Leibler entropy (see e.g., [53]). 

We give preference to the extended thermodynamics of Tsallis that is reviewed in Section 4. This thermodynamic theory has turned out to be effective in many fields, also of applied physics. Only a few examples can be given here. For example, Weberszpil and Chen [54] apply fractional *q*-deformed derivatives to the second law of thermodynamics and generalize, for example, the Maxwell relations to be adapted to non-equilibrium thermodynamics. Authors, as Hamza, Krim and Mohamed, work on medical image registration by maximizing a Tsallis entropy-based divergence (see [55,56]). A multifractal dimensional dependence assessment based on Tsallis mutual information was applied to the structural dynamics of a seismic real series [57]. Beck successfully describes statistical properties, like spatial correlation functions, of fully developed hydrodynamic turbulence by using methods from Tsallis statistical mechanics [58]. Many more applications are summarized in Ref. [59] on the fields of high energy physics, condensed matter physics, astrophysics, geophysics and also lattice Boltzmann models of fluids, turbulence and defect turbulence in Rayleigh-Bénard flows, etc.

## 2. A Brief Review of Some Essentials of the Gibbs-Boltzmann Thermodynamics 

Scientists well aware of Gibbs-Boltzmann and Tsallis extended thermodynamics may skip this section and also the entire Section 4. We start with standard or equilibrium thermodynamics, often referred to as statistical thermodynamics of the Boltzmann-Gibbs (BG) type (see e.g., [60,61,62], etc.). These formulations were applied with success to numerous physical systems in thermal equilibrium, e.g., dilute gases, Boson gases, photon radiation, phonons in solids, fermions and ferromagnetic magnons at low temperatures, etc. (e.g., [61,63], etc.).

The systems, studied in this section, exhibit order/disorder phenomena. High order is described by small entropy and, vice versa. We support the viewpoint that applying the right entropic functional is essential for physical modelling (see [59]). The entropy associated to a BG physical system was proposed by Boltzmann [34] and refined by Gibbs [35] for general systems by the entropy:

(1) $$S_{BG}=-k\sum_{i=1}^{N}p_{i}\log_{e}p_{i},$$

where the $p_{i}$ denotes the microscopic probabilities of the system occupying a partial region $\Omega_{i}$ (1≤ *i ≤ N*) of the total phase space Ω. The quantity *k* is a constant. In case of standard Boltzmann-Gibbs thermodynamics this is the Boltzmann constant *k =* $k_{B}$. Here, we denote it by *k* in reference to the generalizations to nonlinear dynamical systems, where it takes other values. These probabilities obey the normalization:

(2) $$\sum_{i=1}^{N}p_{i}=1.$$

For equal probabilities $p_{i}$ = 1/*N* one derives:

(3) $$S_{BG}=k\log_{e}N.$$

When two probabilistic independent subsystems A and B, with numbers of states $N_{A}$ and $N_{B}$, are put in contact, the joint probabilities obey the special relation:

(4) $$p_{ij}^{A+B}=p_{i}^{A}p_{j}^{B},\forall i,j.$$

In this case the entropy functional (compare with Equation (1)) is:

(5) $$\begin{array}{l} S_{BG}\left(A+B\right)=-k\sum_{i=1}^{N_{A}}\sum_{j=1}^{N_{B}}p_{ij}^{A+B}\log_{e}p_{ij}^{A+B}\\ =-k\sum_{i=1}^{N_{A}}\sum_{j=1}^{N_{B}}p_{i}^{A}p_{j}^{B}\log_{e}\left(p_{i}^{A}p_{j}^{B}\right)\\ =-k\sum_{i=1}^{N_{A}}\sum_{j=1}^{N_{B}}p_{i}^{A}p_{j}^{B}\left(\log_{e}p_{i}^{A}+\log_{e}p_{j}^{B}\right)\\ =-k\left(\sum_{j=1}^{N_{B}}p_{j}^{B}\right)\sum_{i=1}^{N_{A}}p_{i}^{A}\log_{e}p_{i}^{A}-k\left(\sum_{i=1}^{N_{A}}p_{i}^{A}\right)\sum_{j=1}^{N_{B}}p_{j}^{B}\log_{e}p_{j}^{B}. \end{array}$$

By applying Equation (2) to the two parentheses in Equation (5), this simplifies to:

(6) $$S_{BG}\left(A+B\right)=-k\sum_{i=1}^{N_{A}}p_{i}^{A}\log_{e}p_{i}^{A}-k\sum_{j=1}^{N_{B}}p_{j}^{B}\log_{e}p_{j}^{B}.$$

With the help of Equation (1), it follows that the Boltzmann-Gibbs entropy of a joint system A + B with independent subsystems A and B is additive, viz.:

(7) $$S_{BG}\left(A+B\right)=S_{BG}\left(A\right)+S_{BG}\left(B\right).$$

Furthermore, the BG entropy is at its maximum at equal probabilities, shows expansibility to new states and concavity (for proofs of these features see e.g., Tsallis [59,64]).

Consider a system in thermal equilibrium with phase space Ω. For a microscopic or at least a very small part of this system, with a phase space element $\Omega_{i}$, with some simplifying assumptions, the probabilities are given by:

(8) $$p_{i}=\frac{e^{-\beta \;E_{i}}}{Z_{BG}},$$

(see for example [7]). *E_i_* is the energy related to the domain $\Omega_{i}$ of the system. This expression is called Boltzmann factor. Furthermore, the cited derivation yields:

(9) $$\beta =\frac{1}{kT},$$

with the Boltzmann constant *k*, the Kelvin temperature *T* and the partition function of Boltzmann-Gibbs type, given by:

(10) $$Z_{BG}=\sum_{i=1}^{N}e^{-\beta \;E_{i}}.$$

## 3. Kraichnan’s BG-Equilibrium Thermodynamics of *2-d* and *3-d* Turbulent Flow Fields

Now, the question is: can these thermodynamic laws describe turbulence? Kraichnan saw a kind of analogy between a weakly coupled Boson gas below the Bose-Einstein condensation temperature threshold in thermal equilibrium and turbulence. He stated [65]: *There is a fairly close dynamical analogy in which the number density and the kinetic energy of the Bosons play the roles of kinetic energy and squared vorticity*, which is the specific enstrophy of a two-dimensional turbulent field, see [66,67]. We state that this early introduced example is one of the most instructive ones to show how thermodynamics principally applies to turbulence.

From now on, we assume the fluid to be incompressible. Let us start with the divergence of the momentum equation for such a fluid (in the following we mainly follow [68]):

(11) $$div\;\left[\frac{\partial \vec{u}}{\partial t}+\left(\vec{u}\cdot \nabla \right)\;\vec{u}-\nu \;\Delta \vec{u}+\frac{1}{\rho }\nabla p-\vec{f}\right]=0.$$

This transforms to:

(12) $$\frac{\partial }{\partial t}div\;\vec{u}+div\;\left[\left(\vec{u}\cdot \nabla \right)\;\vec{u}\right]-\nu \;\Delta \;div\;\vec{u}+\frac{1}{\rho }\Delta p-div\;\vec{f}=0,$$

which, owing to incompressibility, $div\;\left(\vec{u}\right)=0$, simplifies to the relation:

(13) $$div\;\left[\left(\vec{u}\cdot \nabla \right)\;\vec{u}\right]+\frac{1}{\rho }\Delta p-div\;\vec{f}=0.$$

For a divergence-free specific force field, $div\;\left(\vec{f}\right)=0$, it follows that:

(14) $$\frac{1}{\rho }\Delta p=-div\;\left[\left(\vec{u}\cdot \nabla \right)\;\vec{u}\right].$$

Furthermore, it is evident that:

(15) $$div\;\left[\left(\vec{u}\cdot \nabla \right)\;\vec{u}\right]=\sum_{i,j}^{3}\frac{\partial }{\partial x_{i}}\left(u_{j}\frac{\partial u_{i}}{\partial x_{j}}\right)=\sum_{i,j=1}^{3}\left(\frac{\partial u_{j}}{\partial x_{i}}\frac{\partial u_{i}}{\partial x_{j}}+u_{j}\frac{\partial^{2}u_{i}}{\partial x_{i}x_{j}}\right)=\sum_{i,j=1}^{3}\frac{\partial u_{j}}{\partial x_{i}}\frac{\partial u_{i}}{\partial x_{j}}.$$

Thus, Equation (14) becomes:

(16) $$\frac{1}{\rho }\Delta p=-\sum_{i,j=1}^{3}\frac{\partial u_{j}}{\partial x_{i}}\frac{\partial u_{i}}{\partial x_{j}}.$$

Because the Laplace operator of the pressure is expressible as $\int_{\Gamma }\Delta pdV=\int_{\Gamma }div\;(\nabla p)\;dV$, the integral over a domain Γ, with boundary ∂Γ and for smooth pressure *p*, can be transformed by the divergence theorem to:

(17) $$\int_{\Gamma }\Delta p\;dV=\int_{\Gamma }div\left(\nabla p\right)\;dV=\int_{\partial \Gamma }\frac{\partial p}{\partial \vec{n}}\;d\vec{S},$$

in which $\vec{n}$ is the unit normal vector on ∂Γ pointing to the outside of the domain Γ.

For a Neumann problem, for which:

(18) $$\Delta p\;=-\rho \sum_{i,j=1}^{3}\frac{\partial u_{j}}{\partial x_{i}}\frac{\partial u_{i}}{\partial x_{j}}$$

is solved in Γ subject to the flux boundary condition:

(19) $$\frac{\partial p}{\partial n}=\nabla p\cdot \vec{n},$$

prescribed on ∂Γ, we regard the right-hand side of Equation (18) as prescribed in Γ and know that this so-called Neumann problem for the Poisson equation is mathematically well posed and solvable. The view point given by Equations (18) and (19) allows us to infer that the pressure *p* is a quadratic functional of the fluid velocity field $\vec{u}$. Therefore, we have learnt that in an incompressible fluid the pressure *p* at each instant of time *t* is fully determined by the total velocity field $\vec{u}$ in the (entire) fluid domain and its initial pressure distribution $p_{0}$ at time $t_{0}$, where the velocity field takes the form ${\vec{u}}_{0}$, and so:

(20) $$\frac{p}{\rho }=\psi \left(\vec{u}\right),\frac{p_{0}}{\rho }=\psi \left({\vec{u}}_{0}\right),$$

in which *ψ* is a differentiable function. We, thus, can define the quadratic operator:

(21) $$Β\left(\vec{u},\vec{u}\right)=-\left(\vec{u}\cdot \nabla \right)\;\vec{u}-\nabla \psi \left(\vec{u}\right),$$

so that the momentum equation (see the expression in square brackets of Equation (11)) can be written as:

(22) $$\frac{\partial \vec{u}}{\partial t}-Β\left(\vec{u},\vec{u}\right)-\nu \;\Delta \vec{u}=\vec{f}.$$

Foias et al. [68] write: This equation is not the usual form of the Navier-Stokes equation, but it puts in evidence the fact that those equations may be regarded as functional evolution equations for the velocity $\vec{u}$.

Kraichnan [69] (see also [3,65,70,71,72]) studied the force-free Euler equations in this sense as:

(23) $$\frac{\partial \vec{u}}{\partial t}=Β\left(\vec{u},\vec{u}\right).$$

However, he additionally applied a spectral Ritz-Galerkin truncation with a filtering operator that in the following shall be outlined in detail.

We consider the NSE with periodic boundary conditions, e.g., the solutions of the equation in square brackets of Equation (11) with boundary conditions that are spatially periodic and for simplicity assume that they have the maximum period *L* in each of the three directions. Then, a Fourier series expansion of the velocity is:

(24) $${\vec{u}}^{\left(\kappa^{\prime }\right)}\left(\vec{x}\right)=\sum_{n_{1},n_{2},n_{3}=1}^{+\infty }{\hat{\vec{u}}}_{\vec{n}}^{\left(\kappa^{\prime }\right)}\;e^{\frac{2\pi \;i}{L}\vec{n}\cdot \vec{x}},$$

with the dimensionless integer wave number vector $\vec{n}=(n_{1},n_{2},n_{3})$ and the physical wave number vector $\vec{k}=\kappa^{\prime }(n_{1},n_{2},n_{3})$ in which $\kappa^{\prime }\;\mathrm{has}\;\mathrm{dimension}\;\frac{1}{\mathrm{length}}.$ In particular, the following conditions hold:

(25) $${\hat{\vec{u}}}_{\vec{k}}\cdot \vec{k}=0,{\hat{\vec{u}}}_{-\vec{k}}={\bar{\hat{\vec{u}}}}_{\vec{k}}.$$

The lowest component of the wave number is:

(26) $$\kappa^{\prime }=\frac{2\pi }{L}.$$

If we now cut the spectrum also at a high wave number:

(27) $$\kappa^{\prime \prime }=\frac{2\pi }{L}N,$$

then it follows for the two-fold truncated velocity vector that:

(28) $${\vec{u}}^{\left(\kappa^{\prime },\kappa^{\prime \prime }\right)}\left(\vec{x}\right)=\sum_{n_{1},n_{2},n_{3}=1}^{N}{\hat{\vec{u}}}_{\vec{n}}^{\left(\kappa^{\prime },\kappa^{\prime \prime }\right)}\;e^{i\kappa^{\prime }\;\vec{n}\cdot \vec{x}}.$$

For more details on the truncation procedure, cutting away eddies of higher wave numbers than that of a predefined threshold value *κ*″ ($n_{i}$ > *N*, *i* = 1, 2, 3), respectively setting their Fourier components equal to zero, see also [70,73,74]. This is tantamount to eliminating all Fourier components with wave numbers larger than *κ*″, a fact that is reminiscent of introducing the Kolmogorov dissipation length $l_{K}$, if we assume that *κ*″ is the wave number closest or identical to this inverse dissipation length:

(29) $$\kappa^{\prime \prime }≅\frac{1}{l_{K}}.$$

Galerkin-Ritz spectral procedures project a vector from the entire vector space onto a space spanned by the *N* first eigenvectors (of the Stokes operator, for a definition using duality see [68]), which is orthonormal:

(30) $$\left({\vec{w}}_{n}\cdot {\vec{w}}_{m}\right)=\delta_{n\;m},i\in \left\{1,2,3\right\}.$$

Any velocity vector $\vec{u}$ may be written by means of the vectors of an orthonormal basis and the corresponding Fourier components ${\hat{u}}_{n}:$

(31) $$\vec{u}=\sum_{n=1}^{\infty }{\hat{u}}_{n}{\vec{w}}_{n}.$$

**Figure 1:**
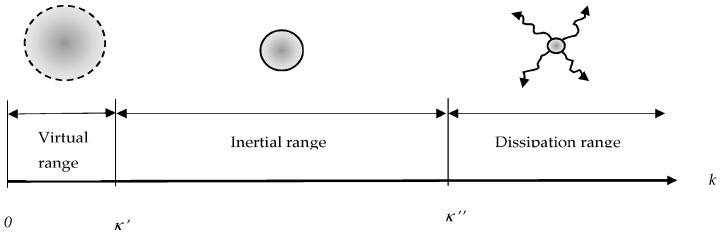
Three domains are distinguished: a virtual range that belongs to virtual eddies of diameter larger than the fluid basin (range to the left), the so-called inertial range with the full ensemble of eddies with a diameter *L* down to $l_{K}$ and the dissipation range with eddies that would have an eddy diameter smaller than the Kolmogorov dissipation scale if they would exist. The circular objects characterize eddies of typical sizes of their domains. The standard Ritz Galerkin truncation neglects wave numbers larger than the bounding wave number value *κ*″.

As described above, a Ritz Galerkin procedure cuts away the highest modes. In a modification we demand that in the summation (31) it removes both, lowest and largest modes (see Figure 1). A projection is described by:

(32) $$P_{N}\vec{u}=\vec{\tilde{u}}=\sum_{n=1}^{N}\left(\vec{\tilde{u}}\cdot {\vec{w}}_{n}\right)\;{\vec{w}}_{n}=\sum_{n=1}^{N}{\hat{u}}_{n}{\vec{w}}_{n},{\hat{u}}_{n}=\left(\vec{\tilde{u}}\cdot {\vec{w}}_{n}\right).$$

A Ritz Galerkin truncation can be interpreted as a projection method to convert a continuous operator problem, e.g., an infinite set of differential equations into a discrete problem, in our case a finite set of evolution equations describing a dynamical system (see [75]). 

Then, a quadratic series development is introduced (compare similarity with the definition in [70]):

(33) $${\tilde{u}}_{i}^{2}={\dot{\tilde{x}}}_{i}^{2}=\sum_{m,n=1}^{N}A_{imn}{\tilde{x}}_{m}{\tilde{x}}_{n},i\in \left\{1,2,3\right\},$$

where here the terms ${\tilde{x}}_{i}$ and ${\tilde{u}}_{i}$ represent independent Cartesian replicas of the space and velocity fields and the terms $A_{imn}$ are spectral coupling coefficients. In this projection operation the constraint:

(34) $$\frac{\partial {\tilde{u}}_{i}}{\partial {\tilde{x}}_{i}}=0,\;(\mathrm{summation}\;\mathrm{over}\;i)$$

remains valid, because the Galerkin truncation operator $P_{n}$ commutes with derivatives. This is a modified interpretation of Kraichnan’s method to account for the fluid incompressibility. 

It follows that Kraichnan’s *2-d* and *3-d* systems conserve each a subspace, namely in the two-dimensional problem defined by the *2-d* kinetic energy per unit of surface mass *ρ*′ (kg m^−2^):

(35) $$E_{\varepsilon }=\frac{1}{2}\rho^{\prime }\int_{\Gamma }{\vec{u}}^{2}\;dA$$

and the enstrophy:

(36) $$\Omega_{\varepsilon }=\varepsilon =\int_{\Gamma }{\vec{\omega }}^{2}dA,\vec{\omega }=\nabla \times \vec{u},$$

whereas, in the three-dimensional case, it is the *3-d* kinetic energy:

(37) $$E_{H}=\frac{1}{2}\rho \int_{\Gamma }{\vec{u}}^{2}\;dV,$$

and the helicity:

(38) $$\Omega_{H}=Η=\int_{\Gamma }\vec{u}\cdot \vec{\omega }\;dV.$$

For brevity reasons, we introduced a different notation, namely the substitutions given by $\Omega_{\varepsilon }=$ *ε*, $\Omega_{Η}=Η$, $E_{\varepsilon }$ and $E_{Η}$.

It is clear that if *κ*″ tends to infinity that a transformation from the discrete to the continuous case, can be performed by replacing sums by corresponding integrals.

**Remark** **1.** *The enstrophy definition which in Equation (36) has been introduced is a special case where incompressibility is assumed to hold. The more general definition of enstrophy is*:

(39) $$\Omega_{\varepsilon }=\int_{\Gamma }{\left|\frac{\partial u_{i}}{\partial x_{j}}\right|}^{2}dA,$$

*where* |…| *denotes the Frobenius norm, which is the sum of the absolute values of all the Jacobian matrix elements* $J_{i,j}=\partial u_{i}/dx_{j},\;i\in \left\{1,2,3\right\}$.

Now, following Kraichnan and going back to the discrete description, one distinguishes: 

(i) Energy conservation:

(40) $${\tilde{E}}_{\chi }\propto \sum_{i=1}^{d}{\tilde{u}}_{i}^{2},\chi \in \left\{\varepsilon ,H\right\},d\in \left\{2,3\right\},$$

with *d* = 2 for *χ* = *ε* and *d* = 3 for *χ* = *H* (we wrote “∝” to neglect the constant *ρ*/2 in this and the following equations).

With Equations (30) and (32) one derives:

(41) $$\begin{array}{ll} {\tilde{E}}_{\chi }\propto & \sum_{i=1}^{d}\sum_{m=1}^{N}{\hat{u}}_{m}w_{im}\sum_{n=1}^{N}{\hat{u}}_{n}w_{in}=\sum_{i=1}^{d}\sum_{m,n=1}^{N}{\hat{u}}_{m}{\hat{u}}_{n}w_{im}w_{in}=\sum_{m,n=1}^{N}{\hat{u}}_{m}{\hat{u}}_{n}\sum_{i=1}^{d}w_{im}w_{in}=\\ & \sum_{m,n=1}^{N}{\hat{u}}_{m}{\hat{u}}_{n}\left({\vec{w}}_{m}\cdot {\vec{w}}_{n}\right)=\sum_{m,n=1}^{N}{\hat{u}}_{m}{\hat{u}}_{n}\delta_{mn}=\sum_{n=1}^{N}{\hat{u}}_{n}^{2}. \end{array}$$

Note the difference in the summation indices of Equations (41)!

(ii) Enstrophy conservation:

(42) $${\tilde{\Omega }}_{\varepsilon }=\sum_{i=1}^{2}{\tilde{\Omega }}_{\varepsilon i}=\sum_{i,j=1}^{2}{\left|\frac{\partial {\tilde{u}}_{i}}{\partial x_{j}}\right|}^{2}.$$

Advantageous is that the operations “taking the derivatives and applying the Ritz-Galerkin projection” commute. 

We start with Equation (24) and in the following auxiliary calculation we omit for brevity reasons the tilde superscript in the velocity components:

(43) $$\frac{\partial u_{i}}{\partial x_{j}}=\frac{\partial \sum_{n_{1},n_{2},n_{3}=1}^{\infty }{\hat{u}}_{i\vec{n}}\;e^{i\;\kappa^{\prime }\;\vec{n}\cdot \vec{x}}}{\partial x_{j}}=i\kappa^{\prime }n_{j}\sum_{n_{1},n_{2},n_{3}=1}^{\infty }{\hat{u}}_{i\vec{n}}\;e^{i\kappa^{\prime }\vec{n}\cdot \vec{x}}=i\kappa^{\prime }n_{j}u_{i}.$$

Furthermore, denoting conjugate complex numbers by overbars, it follows (using Einstein’s summation convention) that:

(44) $${\left|\frac{\partial u_{i}}{\partial x_{j}}\right|}^{2}=\frac{\partial u_{i}}{\partial x_{j}}\bar{\frac{\partial u_{i}}{\partial x_{j}}}=\left(i\kappa^{\prime }n_{j}u_{i}\right)\left(-i\kappa^{\prime }n_{j}u_{i}\right)=\kappa^{{}^{\prime }2}n_{j}n_{j}u_{i}u_{i}.$$

With the discrete version of Equations (39) and (44), it is concluded that:

(45) $$\Omega_{\varepsilon }=\kappa^{{}^{\prime }2}\left(n_{1}^{2}{\tilde{u}}_{1}^{2}+n_{1}^{2}{\tilde{u}}_{2}^{2}+n_{2}^{2}{\tilde{u}}_{1}^{2}+n_{2}^{2}{\tilde{u}}_{2}^{2}\right)=\kappa^{{}^{\prime }2}\left(n_{1}^{2}+n_{2}^{2}\right)\;\left({\tilde{u}}_{1}^{2}+{\tilde{u}}_{2}^{2}\right)=\kappa^{{}^{\prime }2}{\left|\vec{n}\right|}^{2}{\left|\vec{\tilde{u}}\right|}^{2}$$

or:

(46) $$\Omega_{\varepsilon }={\vec{k}}^{2}{\left|\vec{\tilde{u}}\right|}^{2}.$$

Next, we again apply the Ritz-Galerkin truncation and Equations (44)–(46) and obtain:

(47) $${\tilde{\Omega }}_{\varepsilon }\propto {\left|\frac{\partial P_{N}u_{i}^{\left(\kappa^{\prime }\right)}}{\partial x_{j}}\right|}^{2}=\frac{\partial P_{N}u_{i}^{\left(\kappa^{\prime }\right)}}{\partial x_{j}}\bar{\frac{\partial P_{N}u_{i}^{\left(\kappa^{\prime }\right)}}{\partial x_{j}}}={\vec{k}}^{2}{\left(P_{N}u_{i}^{\left(\kappa^{\prime }\right)}\right)}^{2}={\vec{k}}^{2}\sum_{i=1}^{d}{\tilde{u}}_{i}^{2}.$$

With the analogous procedure as described by Equations (40) and (41), we can now derive for the enstrophy the final representation:

(48) $${\tilde{\Omega }}_{\varepsilon }=k^{2}{\hat{\tilde{u}}}^{2}=k^{2}\sum_{n=1}^{N}{\hat{u}}_{n}^{2},$$

where $k=\left|\vec{k}\right|$ denotes the modulus of the wave number $\vec{k}$.

(iii) Helicity conservation:

(49) $${\tilde{\Omega }}_{Η}=k\;{\vec{\tilde{u}}}^{2}=k\sum_{n=1}^{N}\;{\hat{u}}_{n}{}^{2}.$$

Because in this article we are mainly concerned with *2-d* turbulence, we shall not prove representations (49). The *3-d* case shall be treated in future work elsewhere.

Notice that all these conserved quantities are quadratic forms of the velocity vector. However, only for the Euler equation they are strictly conserved. Then, for the discretized version, Kraichnan introduced a set of probability densities by:

(50) $$p_{\chi n}=\frac{e^{-\left(\alpha {\tilde{E}}_{\chi n}+\beta \;{\tilde{\Omega }}_{\chi n}\right)}}{Z_{BG\chi }},\;Z_{BG\chi }=\sum_{n=1}^{N}e^{-\left(\alpha {\tilde{E}}_{\chi n}+\beta \;{\tilde{\Omega }}_{\chi n}\right)},\chi \in \left\{\varepsilon ,H\right\},$$

with the partition function $Z_{BG\chi }$. The generalized energy ${\overset{⌣}{E}}_{\chi }$, and its average quantity $<{\overset{⌣}{E}}_{\chi }>$, are now defined as:

(51) $${\overset{⌣}{E}}_{\chi }=\alpha \;{\tilde{E}}_{\chi }+\beta \;{\tilde{\Omega }}_{\chi },\;\;<{\overset{⌣}{E}}_{\chi }>=\sum_{n=1}^{N}p_{\chi n}\;{\overset{⌣}{E}}_{\chi n}=\frac{\sum_{n=1}^{N}{\overset{⌣}{E}}_{\chi n}\;e^{-{\overset{⌣}{E}}_{\chi n}}}{Z_{BG\chi }}.$$

Because in Equations (40), (46), (48) and (49), ${\tilde{E}}_{\chi }$ and ${\tilde{\Omega }}_{\chi }$ are quadratic functions of ${\tilde{u}}_{i}$ or ${\hat{u}}_{n}$, we know that this system of evolution equations, constituting a conservative dynamical system, is obeying Gaussian statistics.

A Fourier transform of ${\overset{⌣}{E}}_{\varepsilon }$ for *2-d* turbulence leads to the following generalized energy-enstrophy spectrum (see Refs. [3,70] and for its derivation the Appendix A, Equation (A34)):

(52) $$2-d:\;{\hat{\overset{⌣}{E}}}_{\varepsilon }\left(k\right)=\frac{1}{2}\frac{1}{\alpha +\beta \;k^{2}},$$

where *α* and *β* are constants. On the other hand, a Fourier transform of ${\overset{⌣}{E}}_{H}$ for *3-d* turbulence leads to the energy spectrum (see also [3,70]):

(53) $$3-d:\;{\hat{\overset{⌣}{E}}}_{H}\left(k\right)\propto k^{2},$$

in which ${\hat{\overset{⌣}{E}}}_{H}(k)$ is the Fourier transform of ${\overset{⌣}{E}}_{H}(k)$. Kraichnan calls expressions in Equations (52) and (53) the ‘mean modal intensity spectra’. Then, the ‘isotropic energy and enstrophy spectrum’ of *2-d* turbulence follows by Equations (54). The second formula is only valid, if one is dealing with power laws:

(54) $$S_{\chi }\left(k\right)=\frac{d{\hat{\overset{⌣}{E}}}_{\chi }}{dk}\propto \frac{1}{k}{\hat{\overset{⌣}{E}}}_{\chi },\chi \in \left\{\varepsilon ,H\right\}.$$

In the *2-d* situation Equation (52) implies:

(55) $$k\ll \sqrt{\frac{\alpha }{\beta }}\Rightarrow {\hat{\overset{⌣}{E}}}_{\varepsilon }(k)=\frac{1}{2\alpha }=\mathrm{const}\;\mathrm{and}\;k\gg \sqrt{\frac{\alpha }{\beta }}\Rightarrow {\hat{\overset{⌣}{E}}}_{\varepsilon }(k)=\frac{1}{2\beta }\frac{1}{k^{2}}.$$

By applying Equation (54), the energy and enstrophy spectra for the low and high wave number regimes are:

(56) $$k\ll \sqrt{\frac{\alpha }{\beta }}\Rightarrow S_{\varepsilon }(k)\equiv 0\;\mathrm{and}\;k\gg \sqrt{\frac{\alpha }{\beta }}\Rightarrow S_{\varepsilon }(k)\propto \frac{1}{k^{3}}.$$

Whereas the energy spectrum shows the useless value zero, the enstrophy spectrum is in excellent agreement with computer experiments (see Section 8 below). This suggests that at high wave numbers the occurring small eddies are close to or even in thermodynamic equilibrium. However, the experimentators state that their simulation results may be slightly erroneous because of aliasing and slow convergence of their calculations. This is motivation for us to also *q*-generalize the results for the large wave number regime. In future, if even higher precision experiments would be available, the power law exponent of this spectrum could be adjusted with a small non-equilibrium contribution. In any case *q* values very close to “1”, respectively power law exponents close to “−3” are expected to occur. Kraichnan called the high wave number regime ‘inverse cascade’ of two-dimensional turbulence ([65,76,77]).

We ask ourselves: What is the essential conclusion of this instructive spectral example, derived by Kraichnan fifty years ago? The answer to this question is given by:

**Proposition** **1.** *By a Ritz-Galerkin truncation of the 2-d and 3-d Euler equations, these equations can be transformed to a finite system of evolution equations with two conserved quadratic forms that obey Gaussian statistics*.

Furthermore, we have corroborated and now state:

**Proposition** **2.** *Truncating the momentum equation and applying Boltzmann-Gibbs equilibrium statistical mechanics (thermodynamics) leads to a loss of essential features of low-wave number turbulence. It is a too bold approach of modeling turbulent fields, like the application of a linear and local closure scheme* (see Table 1).

Today it is known that the entropy functional and the partition function, respectively, define ‘universality classes’ showing identical ‘critical exponents’ (see [31]). This means that a system close to its criticality exhibits a kind of universal behavior that is not determined by the specific microscopic interactions of neighboring particles. It was shown, for instance, that a liquid-gas transition and a ferromagnetic system (lattice gas and Ising model), in the region just below criticality, possess with high precision the same scaling exponents, because they belong to the same universality class (Refs. [29,30]). This agrees with the statement of Goldenfeld [27] that phenomena with the same set of critical exponents are said to form a universality class. Therefore, one ought to distinguish between exact equivalences, where two models are analogous to one another (with a unique mapping from one to the other) and approximate equivalences, in which the partition functions may be (slightly) different, but nevertheless comprise two systems that show the same or similar behavior near criticality. According to Goldenfeld [27] members of the same universality class have the following three properties in common:

(1) The symmetry group of the Hamiltonian,
(2) The dimensionality of the physical problem,
(3) The range of the forces (short or long).

With these facts, we assert that the Boltzmann-Gibbs thermodynamics is inadequate to describe turbulence sufficiently accurately. In nonlinear dynamics (see [53]) different approaches have been worked out, which are also presented in Tsallis [59]. They involve different entropy functionals, as e.g., the Normalized entropy, Shannon entropy, Rényi entropy, Kolmogorov-Sinai entropy, Escort entropy, etc. Up-to-present, not all questions have been answered concerning what kind of generalized entropy functional is optimal and which one best applies to a specific complex system.

## 4. An Introduction to the Extended Thermodynamics of Tsallis 

Below we demonstrate that yet a further entropy functional, called Tsallis entropy, is particularly optimal to describe turbulence. This entropy can be directly related to fractal geometry and Lévy flight statistics (see [78,79]). Furthermore, the above class of entropies have many features in common and the Tsallis entropy enjoys simple relations with other entropies listed e.g., in [59].

Let us introduce the Tsallis entropy in a metaphoric manner (see [59]). To this end, we aim for the derivation of Equation (3) by considering the linear differential equation:

(57) $$\frac{dN}{dS_{BG}}=\frac{1}{k}N,N(0)=1,$$

in which, for simplicity, we concentrate on the case of a single probability, i.e., *N* equals “1”; for this case:

(58) $$p=\frac{1}{N}\Leftrightarrow S_{BG}=<S_{BG}>.$$

Writing (57) as:

(59) $$\frac{dN}{N}=d\left(\log_{e}N\right)=\frac{1}{k}dS_{BG},$$

its integration yields:

(60) $$\log_{e}N=\frac{1}{k}S_{BG}+\log_{e}C,$$

in which $\log_{e}C$ is the constant of integration. It follows that:

(61) $$N(S_{BG})=Ce^{\frac{1}{k}S_{BG}}.$$

With Equation (60) the constant *C* is determined by expressing the requirement of certainty (which is equivalent to $S_{BG}(1)=0$) as:

(62) $$S_{BG}\left(1\right)=k\log_{e}\left(1\right)-k\log_{e}\left(C\right)=0,$$

implying *C* = 1, so that, owing to Equation (60):

(63) $$\langle S_{BG}\rangle \;(N)=k\log_{e}N,$$

which is also the entropy of a system with elements having equal probabilities that was already anticipated by Equation (3). With Equations (58) and (63) this is generalized to become:

(64) $$S_{BG}(N)=p\langle S_{BG}\rangle =-k\;p\log_{e}p,$$

(compare with Equation (1) for a single state *p*) for a system of *N* elements with the single element probability *p*. Furthermore, Equation (61) reduces to:

(65) $$N(S_{BG})=e^{\frac{1}{k}S_{BG}}.$$

At this stage the differential equation with its initial condition (57), is suggestive for a generalization of the Boltzmann-Gibbs entropy functional to nonlinearity. Following this recipe, Tsallis proposed a nonlinear scaling with value q∈ℝ of the linear differential Equation (57), which is:

(66) $$\frac{dN}{dS_{q}}=\frac{1}{k}N^{q},N(0)=1.$$

Writing (66) alternatively as:

(67) $$\frac{dN}{N^{q}}=\frac{1}{1-q}d\;\left(N^{1-q}\right)=\frac{1}{k}dS_{q},$$

a straightforward integration yields the result:

(68) $$N^{1-q}=\left(1-q\right)\frac{1}{k}S_{q}+C,$$

where *C* is a constant of integration, which is determined to be *C* = 1, since for *N* = 1, $S_{q}=0$. Thus, the general integral of (66) is:

(69) $$N\left(S_{q}\right)={\left[1+\left(1-q\right)\frac{1}{k}S_{q}\right]}^{1/\left(1-q\right)}.$$

Now, we shall define a generalization of the exponential function, called ‘*q*-exponential function’ (suggested by Tsallis [59]) and given by Equation (69):

(70) $$e_{q}^{x}:={\left[1+\left(1-q\right)x\right]}^{1/\left(1-q\right)}.$$

**Figure 2:**
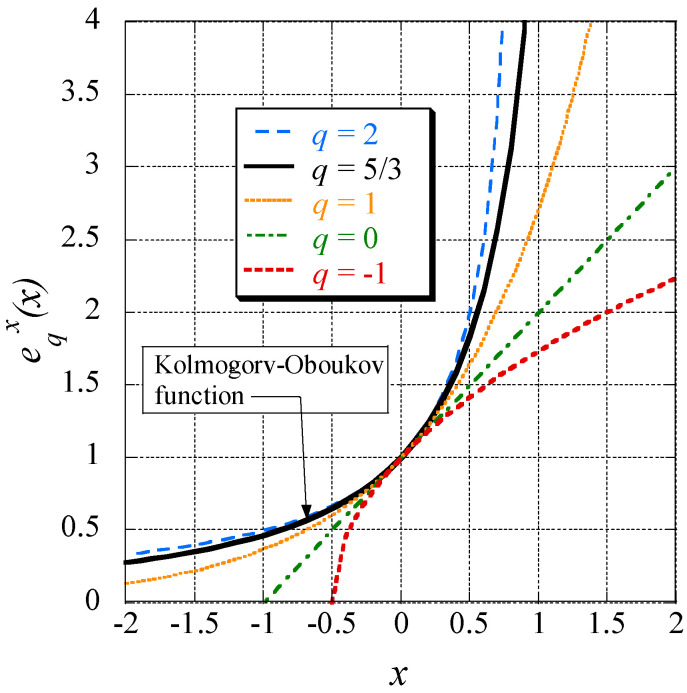
The *q*-exponential function $e_{q}^{x}$ for typical values of *q*. After Tsallis [59], reproduced with additions, e.g., the Kolmogorov-Oboukov function (see Section 5).

This function is displayed in Figure 2 for different values of *q*. In agreement with relation (70) and *n* = 1/(1 − *q*) the well-known formula, defining the usual exponential function, emerges if the limit q→1 of Equation (70) is taken, viz.:

(71) $$e_{1}^{x}=\underset{q\to 1}{\lim}\;{\left[1+\left(1-q\right)x\right]}^{1/\left(1-q\right)}=\underset{n\to \infty }{\lim}\;{\left[1+\frac{x}{n}\right]}^{n}=e^{x}.$$

From Equations (69) and (71), we draw the following inference:

(72) $$N(S_{1})=e^{\frac{1}{k}S_{1}}=e^{\frac{1}{k}S_{BG}}=N(S_{BG}).$$

For *q* = 1, the BG-entropy $S_{BG}$ and the Tsallis entropy $S_{q}$ are the same.

On the other hand, the inverse function of (69) is:

(73) $$S_{q}\left(N\right)=k\frac{1}{1-q}\left(N^{1-q}-1\right).$$

This now suggests the definition of the ‘*q*-logarithmic function’:

(74) $$\ln_{q}\left(x\right):=\frac{1}{1-q}\left(x^{1-q}-1\right),$$

where here, $\log_{e}x=\ln x$ is used to avoid a confusion between the *q* exponent and the basis *b* of the logarithmic function log *b*. The definition (74) also implies:

(75) $$\ln_{q}\left(\frac{1}{x}\right):=\frac{1}{1-q}\left(x^{q-1}-1\right).$$

Moreover, in agreement with Equation (74) and the assignment *n* = 1/(1 − *q*) the formula defining the usual logarithmic function is deduced from the following chain:

(76) $$\ln_{1}\left(x\right)=\underset{q\to 1}{\lim}\frac{1}{1-q}\left(x^{1-q}-1\right)=\underset{n\to \infty }{\lim}\;n\left(x^{1/n}-1\right)=\ln\left(x\right).$$

From the above presented metaphor and Equations (73) and (74), Tsallis, for equal probabilities, postulated the following generalization of Equation (3):

(77) $$S_{q}\left(N\right)=k\ln_{q}\left(N\right).$$

**Figure 3:**
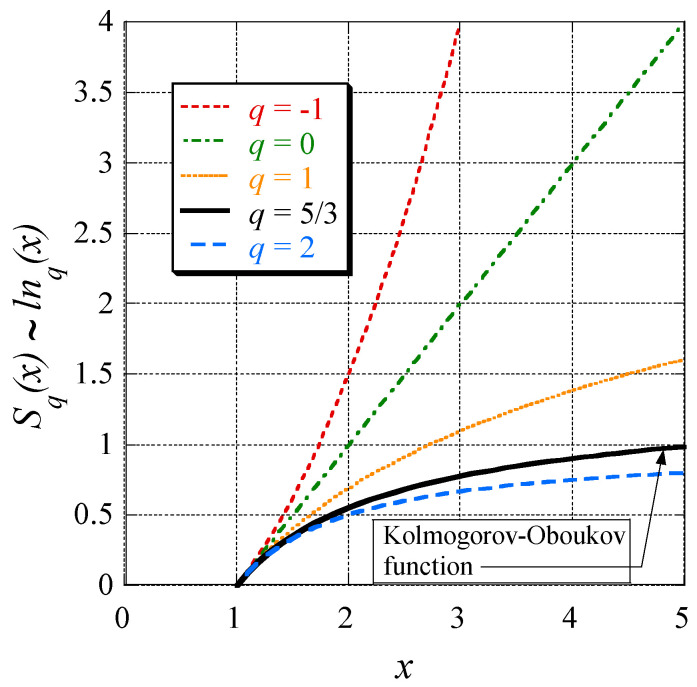
The equiprobability entropic functional $S_{q}\left(x\right)$ plotted against a continuous number of states *x* for *k* = 1 and different parameters *q*. After Tsallis [59] reproduced with changes and additions. e.g, the Kolmogorov-Oboukov function (see Section 5).

In Figure 3 this function is displayed for *k* = 1 and different *q*-values.

With *p* = 1/*N*, $S_{q}$ in Equation (77) can be expressed as a function of *p*:

(78) $$S_{q}\left(p\right)=k\ln_{q}\left(\frac{1}{p}\right).$$

Notice that it must be carefully checked which rules of the usual logarithm apply to its generalized form. The relation ln*_q_*(1/*p*) is not identical to −ln*_q_*(*p*) unless *q* = 1; thus, we cannot write this equation in the *q*-generalized form of Equation (1) for a single probability.

The next step is to demand:

(79) $$S_{q}\left(N\right)=k\left[\ln_{q}\left(\frac{1}{p}\right)\right].$$

In this generalization process, arbitrary probabilities must be introduced as:

(80) $$S_{q}\left(N\right)=k\;\sum_{i=1}^{N}p_{i}\ln_{q}\left(\frac{1}{p_{i}}\right).$$

In agreement with Equation (76), we conclude that, according to (80) and (1) for *N* = 1:

(81) $$S_{1}=k\sum_{i=1}^{N}p_{i}\ln\;\left(\frac{1}{p_{i}}\right)=-k\sum_{i=1}^{N}p_{i}\ln p_{i}=S_{BG},$$

and, therefore, as already stated after Equation (72), equilibrium thermodynamics of the Boltzmann-Gibbs type is a special case of the, thus, proposed Tsallis non-equilibrium thermodynamics.

Inserting definition (75) of the *q*-logarithmic function into (80) leads to:

(82) $$S_{q}\left(N\right)=\frac{k}{1-q}\;\sum_{i=1}^{N}p_{i}\left(p_{i}{}^{q-1}-1\right),$$

which, with the normalization (2), is identical to:

(83) $$S_{q}\left(N\right)=k\;\frac{1-\sum_{i=1}^{N}p_{i}{}^{q}}{q-1}.$$

This *q*-entropy or Tsallis entropy generalizes the entropy of an equilibrium to that of a non-equilibrium thermodynamic system, respectively from systems with linear to those with nonlinear and complex behavior; the latter is suitable for turbulence. Notice that this entropy initially was introduced by ideas of fractal geometry. It follows that:

(84) $$\begin{array}{c} q<1:\Leftrightarrow p_{i}^{q}>p_{i},\\ q=1:\Leftrightarrow p_{i}^{q}=p_{i},\\ q>1:\Leftrightarrow p_{i}^{q}<p_{i}. \end{array}$$

Next, a new set of probabilities is introduced by defining, with index *ES* denoting ”Escort”:

(85) $$P_{i}=\frac{p_{i}^{q}}{Z_{ES}},Z_{ES}=\sum_{i=1}^{N}p_{i}^{q}.$$

The complete set $\left\{P_{i}\right\}$ is called ‘escort probabilities’. They obviously also fulfill the normalization condition:

(86) $$\sum_{i=1}^{N}P_{i}=1.$$

The condition *q* < 1 makes the escort probabilities larger than the $p_{i}{}^{\prime }s$, and *q* > 1 makes them smaller. Therefore, *q* < 1 enhances the occurrence of rare or large-scale events and *q* > 1 enhances those of frequent or small-scale events. We experience here a connection with Lévy statistics in which the case *q* < 5/3 corresponds to sub diffusivity, *q* = 5/3 describes the standard Brown or normal diffusion, obeying Gaussian statistics, and the case *q* > 5/3 belongs to super diffusivity (see Table 2 and Table 3).

We will identify more quantitative relations between Lévy statistics, anomalous diffusion and extensive thermodynamics in Section 5. When *q* < 1, it is important that summations must exclude all probabilities of zero value (because of singular behavior), whereas for *q* > 1 the summation may include all occurring probabilities. 

Next, let us investigate the compositional property of the Tsallis entropy. Then, from Equation (74), we conclude that:

(87) $$\left(1-q\right)\ln_{q}\left(x_{A}x_{B}\right)=\left[{\left(x_{A}x_{B}\right)}^{1-q}-1\right].$$

This is identical to the following extended mathematical expression:

(88) $$\begin{array}{l} {\left(x_{A}x_{B}\right)}^{1-q}-1=\left(x_{A}{}^{1-q}-1\right)+\left(x_{B}{}^{1-q}-1\right)+{\left(x_{A}x_{B}\right)}^{1-q}-x_{A}{}^{1-q}-x_{B}{}^{1-q}+1=\\ \left(x_{A}{}^{1-q}-1\right)+\left(x_{B}{}^{1-q}-1\right)+\left(x_{A}{}^{1-q}-1\right)\left(x_{B}{}^{1-q}-1\right), \end{array}$$

which with use of Equation (87) is now rewritten to take the form:

(89) $${\left(x_{A}x_{B}\right)}^{1-q}-1=\left(1-q\right)\ln_{q}\left(x_{A}\right)+\left(1-q\right)\ln_{q}\left(x_{B}\right)+\left(1-q\right)\ln_{q}\left(x_{A}\right)\;\left(1-q\right)\ln_{q}\left(x_{B}\right).$$

With the help of Equations (73) and (74) these expressions are transformed back to Tsallis entropies. This yields:

(90) $$\frac{S_{q}\left(A+B\right)}{k}=\frac{S_{q}\left(A\right)}{k}+\frac{S_{q}\left(B\right)}{k}+\left(1-q\right)\frac{S_{q}\left(A\right)}{k}\frac{S_{q}\left(B\right)}{k},$$

which demonstrates non-additivity of $S_{q}$ for *q* ≠ 1. Depending on *q*, it follows that:

(91) $$\begin{array}{c} q<1:\;S_{q}\left(A+B\right)>S_{q}\left(A\right)+S_{q}\left(B\right),\\ q=1:\;S_{q}\left(A+B\right)=S_{q}\left(A\right)+S_{q}\left(B\right),\\ q>1:\;S_{q}\left(A+B\right)<S_{q}\left(A\right)+S_{q}\left(B\right). \end{array}$$

**Figure 4:**
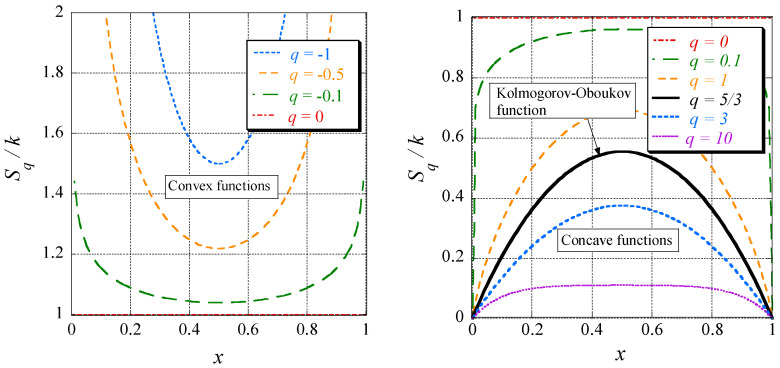
The *q* entropy for *N* = 2 for negative (on the left) and positive *q*-values (on the right). We stress that all *q* entropies are strictly convex or concave, a feature that not all generalized entropies (e.g., Renyi, Escort, etc.) fulfil. After Tsallis [59] reproduced with additions. e.g., the Kolmogorov-Oboukov function (see Section 5).

Therefore, for *q* < 0, $S_{q}(p)$ is a convex function, whereas for equal probabilities, $p_{1}=p=1/2$ and $p_{2}=1-p=1/2$, it takes a minimum (Figure 4, left). On the other hand, for *q* > 0 it is concave with a maximum for equal probabilities in this case (Figure 4, right). Slowly, it becomes transparent that non-extensive thermodynamics is an appropriate framework to tackle anomalous diffusion and, thus, also turbulence (see [80]).

## 5. Relation between Lévy Statistics and Tsallis Extended Thermodynamics

To make the entire situation more transparent, we now outline the connections between Lévy walk and Lévy flight dynamics (see [80,81,82]), respectively, and the Tsallis non-extensive and non-additive thermodynamics (see [59,64]). 

The mean square displacement of diffusive problems is generalized in [80]:

(92) $$\langle x^{2}\left(t\right)\rangle \propto t^{\alpha },$$

with time *t* and exponent α=2H=1, describing Brownian diffusion. *H* denotes the so-called ‘Hurst exponent’.

After Alemany and Zanette [81] the jump probability distribution can be obtained from Equation (1) by maximizing the entropy $S_{BG}$, in the continuous case written as an integral:

(93) $$S_{BG}\left[p\;\left(x\right)\right]=-k\int_{\Gamma }p\left(x\right)\log_{e}\left[p\left(x\right)\right]\;dx,\Gamma \in \mathbb{R},$$

subject to the two auxiliary constraints, namely:

(94) $$\int_{\Gamma }p\left(x\right)\;dx-1=0$$

and:

(95) $$\int_{\Gamma }x^{2}p\left(x\right)\;dx-\sigma^{2}=0;$$

the quantity *σ* is the standard deviation. For simplicity, we have chosen the *1-d* representation. For higher dimensions the considerations are analogous (see [81]). The problem of maximizing the entropy (93), subject to the constraints (94) and (95) is technically performed by maximizing the Lagrange function:

(96) $$\Phi \left[p\;\left(x\right)\right]=-k\int_{\Gamma }p\left(x\right)\log_{e}p\left(x\right)\;dx-\lambda_{1}\left[\int_{\Gamma }p\left(x\right)\;dx-1\right]-\lambda_{2}\left[\int_{\Gamma }x^{2}p\left(x\right)\;dx-\sigma \right],$$

in which $\lambda_{1}$ and $\lambda_{2}$ are the Lagrange parameters. The solution of this extremum principle determines these Lagrange parameters as well as the jump probability distribution, which takes the form:

(97) $$p\left(x\right)=A\exp\left(-\frac{x^{2}}{2\sigma^{2}}\right),$$

where for details it is suggested to consult e.g., the book on fluctuation theory by Montroll and Lebowitz [82]. The proof of Equation (97) is given as a special case of the proof of Equation (103) (paragraph below and [81]).

A Fourier transform of Equation (97) for small wave number *k* (large wave length *λ*) leads to the characteristic function (see [81]) of the following form:

(98) $$G\left(k\right)\approx 1-\frac{1}{2}\sigma^{2}k^{2}+\ldots .$$

With innovative imagination, this is now generalized as:

(99) $$G\left(k\right)\approx 1-\alpha^{2}k^{\gamma_{L}}+\ldots ,\gamma_{L}\in R+,$$

with $\gamma_{L}=2$ characterizing Brownian diffusion (compare with Equations (97) and (98)). An inverse Fourier transform of this equation reveals the asymptotic Lévy distribution appropriate for large distances, with the scaling property:

(100) $$p(x)\;\sim \;x^{-\gamma_{L}-1},\;\mathrm{for}\;\left|x\right|\to \infty .$$

This is called the long tail of the Lévy probability distribution. To be precise, the approximate large distance (small *k*) Fourier transform of (99) is (100), a result of the linear equilibrium theory, which is then made nonlinear in (99), and this approximation in Fourier space is assumed to be representative for the long-tail behaviour of the Lévy probability distribution expressed as Equation (100). 

The same idea of applying an extremum principle and by taking into account generalizations of the constraints (94) and (95), replacing the probability *p* by the escort probability *P*, viz.:

(101) $$\int_{\Gamma }P\left(x\right)\;dx-1=0,\int_{\Gamma }x^{2}P\left(x\right)\;dx-\sigma_{q}^{2}=0,$$

with the quantity $\sigma_{q}^{}$ playing the role of a *q*-generalized standard deviation and with the *q*-entropy $S_{q}$ (see Equation (83)), viz.:

(102) $$S_{q}\left[p\left(x\right)\right]=k\frac{1-{\int_{\Gamma }p\left(x\right)}^{q}\;dx}{q-1},$$

leads to the result (see [81]), with the generalized Boltzmann constant *k*:

(103) $$p\left(x\right)={\left[\frac{kq}{\lambda_{1}\left(q-1\right)}+\frac{\lambda_{2}\;q}{\lambda_{1}}x^{2}\right]}^{1/\left(1-q\right)},$$

and the two Lagrange multipliers $\lambda_{1}$ and $\lambda_{2}$. The applied method is outlined in more detail in the next section and the proof of Equation (103) is given in [79]). Furthermore, more information on the Lagrange multipliers is given in Section 10.

For large distances *x*, setting the exponents of the formulas Equations (100) and (103) equal to one another, yields:

(104) $$\gamma_{L}=\frac{3-q}{q-1},q=\frac{3+\gamma_{L}}{1+\gamma_{L}}.$$

These two relations directly connect Lévy flight statistics with the thermodynamic formalism of Tsallis. The corresponding characteristic parameters are presented in Table 3.

## 6. Escort Probability Distribution and Expectation Values 

The entropy optimization method, as applied by Kraichnan in 1967 (see [70]) for BG thermodynamics, shall now be analogously applied to the Tsallis thermodynamic theory, because it is a prerequisite to perform thermodynamics with generalized entropic forms. With some small deviations, we follow mainly this reference.

Let us now, in analogy to Equation (85), introduce the continuous escort probability distribution:

(105) $$P(x)=\frac{{\left[p(x)\right]}^{q}}{Z_{ES}},Z_{ES}=\int_{0}^{\infty }{\left[p(x)\right]}^{q}dx,$$

in which the integral is introduced as a Lebesque integral with continuous measure. The distribution is also normalized, viz.:

(106) $$\int_{0}^{\infty }P(x)\;dx=1.$$

Furthermore, by defining the *q*-average value of a function *f* as:

(107) $${\langle f\left(x\right)\rangle }_{q}=\int_{0}^{\infty }f\left(x\right)P\left(x\right)\;dx=F_{q},$$

it follows for *f* = *x*, that:

(108) $${\langle x\rangle }_{q}=\int_{0}^{\infty }xP\left(x\right)\;dx=X_{q},$$

which is the *q*-average value of *x*. 

To optimize the entropy distribution $S_{q}\left[P\right]$, in analogy to Equation (96), we define the Lagrange function Φ, containing the two constraints Equations (106) and (107) for the energy function given by *f*(*x*) = *E*(*x*):

(109) $$\Phi \left[P\right]=S_{q}\left[P\right]-\lambda_{1}\left[\int_{0}^{\infty }P(x)\;dx-1\right]-\lambda_{2}\left[\int_{0}^{\infty }P(x)E\left(x\right)\;dx-E_{q}\right].$$

Substituting the *q* entropy, Equation (102), yields:

(110) $$\Phi \left[P\right]=k\frac{1-\int_{0}^{\infty }\left[P(x)\right]\;dx}{q-1}-\lambda_{1}\left[\int_{0}^{\infty }P(x)\;dx-1\right]-\lambda_{2}\left[\int_{0}^{\infty }P(x)E\left(x\right)\;dx-E_{q}\right],$$

in which $E_{q}$ is the average energy defined below (see Equation (115)) and where the denominator *q*−1 can be taken as a quantitative measure of “Non-BG behavior”. 

Maximization is obtained by performing the optimization dΦ[P]/dP=0; it leads to the optimum result $P_{\max}$, where, upon dropping the index, one obtains (see [59]):

(111) $$P\left[E\left(x\right)\right]=\frac{e_{q}^{-\beta_{q}\;\left[E\left(x\right)\right]}}{\int_{0}^{\infty }e_{q}^{-\beta_{q}\;\left[E\left(x\right)\right]}dx},$$

The optimization process determines the generalized Boltzmann factor P [E(x)], which is called here Tsallis factor. One can show that the Lagrange multiplier $\lambda_{1}$, due to Equation (106), factorizes out (see [59]).

Now, a *q*-generalized partition function is defined by:

(112) $$Z_{q}:\;=\int_{0}^{\infty }e_{q}^{-\beta_{q}\;\left[E\left(x\right)\right]}dx$$

When applying the *q*-exponential function (70) and definition (112), the Tsallis factor (111) is found to be given by (see also in [79]):

(113) $$P\left[E\left(x\right)\right]=\frac{{\left[1+\left(1-q\right)\beta_{q}E\left(x\right)\right]}^{1/\left(1-q\right)}}{Z_{q}}.$$

Next, the average energy is calculated by:

(114) $${\langle E\left(x\right)\rangle }_{q}=E_{q}=\int_{0}^{\infty }P\left[E\left(x\right)\right]\;E\left(x\right)\;dx.$$

Substituting Equation (113) into (114) leads to:

(115) $$E_{q}=\frac{\int_{0}^{\infty }{\left[1+\left(1-q\right)\beta_{q}E\left(x\right)\right]}^{1/\left(1-q\right)}\;E\left(x\right)\;dx}{Z_{q}}.$$

## 7. Fractional Calculus: A Promising Method to Describe Turbulence

In analogy to Kraichnan’s calculus of the energy-enstrophy spectrum, to obtain the *q*-generalized versions of Equations (52), (55) and (56), we require fractional calculus. To be able to generalize the steps between these equations, the Fourier transform of a derivative and its square must be calculated. Therefore, we deal in a general manner with fractional derivatives. The reason is that the common Riemannian derivative, in our case, insufficiently describes turbulent phenomena. The adequate new tools for our problem are fractional derivatives and integrals. Such were, for example, also applied with success by the present authors to turbulence modeling of turbulent shear flows (see e.g., [26]). 

In Fourier calculus the following main principle holds:

(116) $$k^{m}{\left(i\frac{d}{dk}\right)}^{n}\hat{f}\left(k\right)=F\left[{\left(-i\frac{d}{dx}\right)}^{m}x^{n}f\left(x\right)\right],$$

where F denotes the Fourier transform. Now, we study the special case *n* = 0, which yields:

(117) $$F\{{\left(-i\right)}^{m}\frac{d^{m}f\left(x\right)}{dx^{m}}\}=k^{m}\hat{f}\left(k\right).$$

We observe that by a Fourier transform a derivative of order *m* of a function *f(x)* is transformed to a power law of the wave number *k*, with exponent *m*, times the Fourier transformed function. This is the basic rule to generalize the usual derivative to the Fourier fractional derivative of order *m* = *q*:

(118) $$F\{{\left(-i\right)}^{q}\frac{d^{q}f\left(x\right)}{dx^{q}}\}=k^{q}\hat{f}\left(k\right),{\left(-i\right)}^{q}=e^{-i\frac{\pi }{2}q}.$$

The rule for usual integer-order derivatives and power laws with integer exponents, is generalized in fractional calculus to real and complex numbers (see e.g., Refs. [83,84]); in our context real-order derivatives are sufficient. By applying the Fourier fractional derivative to a power law, one finds (see e.g., [84]):

(119) $$\frac{d^{q}}{dx_{q}}x^{\alpha }=\frac{\Gamma \left(q+\alpha \right)}{\Gamma \left(\alpha \right)}x^{\alpha -q},x>0.$$

Now, we set *q* = 1, which yields:

(120) $$\frac{d}{dx}x^{\alpha }=\frac{\Gamma \left(1+\alpha \right)}{\Gamma \left(\alpha \right)}x^{\alpha -1},x>0.$$

With the formula (see [85]):

(121) $$\frac{\Gamma \left(1+\alpha \right)}{\Gamma \left(\alpha \right)}=\alpha ,$$

Equation (119) yields:

(122) $$\frac{d}{dx}x^{\alpha }=\alpha \;x^{\alpha -1},x>0,$$

which is the usual Riemannian differentiation rule for a power law with exponent *α*. In fractional calculus there is no consensus on the differentiation of a constant. There are essentially two definitions:

(123) $$\frac{d^{q}}{\partial x^{q}}const=0\mathrm{and}\frac{d^{q}}{\partial x^{q}}const=c\;x^{0-q}=c\;x^{-q},c=c\left(\Gamma \left(q\right)\right),$$

where definition (123) is due to Caputo’s fractional derivative [84] and Equation (123) is the solution for a Riemannian fractional derivative [84] with a constant *c* being a function of Gamma functions and is motivated by Equation (119) with *α* = 0. As the first definition leads also to the useless result (56, left), derived with BG thermodynamics, the second one is very promising and, therefore, leads us in a slightly pragmatic way to give it preference (see Section 8). 

## 8. Fractional Generalization of Kraichnan’s Spectra and Their Validation by Numerical Experiments

With such knowledge, Equation (54) is now *q*-generalized in the following manner:

(124) $$S_{\varepsilon }\left(k\right)=D_{k}^{\left(q\right)}\hat{\overset{⌣}{E}}\left(k\right)=\frac{d^{q}}{dk^{q}}\hat{\overset{⌣}{E}}\left(k\right).$$

Now, a Fourier transform of ${\overset{⌣}{E}}_{\varepsilon }$ for *2-d* turbulence leads to the following generalized energy-enstrophy spectrum (its proof is outlined in the Appendix A, see especially Equation (A33)):

(125) $${\hat{\overset{⌣}{E}}}_{\varepsilon }(k)=\frac{1}{2}\frac{1}{Z_{ES\varepsilon }}\frac{1}{q^{2}}\frac{1}{\alpha +\beta \;k^{2q}}.$$

Two wave number regions emerge from this formula. 

*The first* is the small wave number regime, for which, from Equation (125), it follows that:

(126) $${\hat{\overset{⌣}{E}}}_{\varepsilon }(k)=\frac{1}{2\alpha }\frac{1}{Z_{ES\varepsilon }}\frac{1}{q^{2}}=const.$$

Now, by applying Equation (123), one calculates the fractional derivative of this constant:

(127) $$S_{\varepsilon }(k)=\frac{1}{2\alpha }\frac{1}{Z_{ES\varepsilon }}\frac{1}{q^{2}}c\left(\Gamma \right)\frac{1}{k^{q}}=const\frac{1}{k^{q}},$$

which is different from Equation (56). Notice that in Equation (127), with *q* = 1, a reciprocal energy spectrum does not result, because in this case *c* = 0 will guarantee agreement with the usual differrentiation, so that (56) also occurs as special case of this new treatment. This would be a wave number spectrum related to the thermodynamic equilibrium of a turbulent flow field at small wave numbers, which does not exist! 

However, as we can see in the third line of Table 4, there is another special case, namely the one corresponding to Brownian diffusion, which is characterized by *q* = 5/3, leading to the well verified Kolmogorov-Oboukov energy spectrum:

(128) $$S_{\varepsilon }\left(k\right)=constk^{-5/3}.$$

This result is in best agreement with computer experiments for *2-d* turbulence performed and reported by Lilly [86] and reviewed by Kraichnan [70]. Let us summarize:

**Proposition** **3.** *Tsallis’ extended thermodynamics generalizes Kraichnan’s constant turbulent energy by a power-law energy spectrum with exponent −q, which relates this generalized spectrum directly with the Kolmogorov-Oboukov −5/3 law and its fractal generalization*, e.g., *given by the fractal β-model* (*see* e.g., *Frisch [3]), describing turbulent flow fields with intermittency (see below)*.

**Figure 5:**
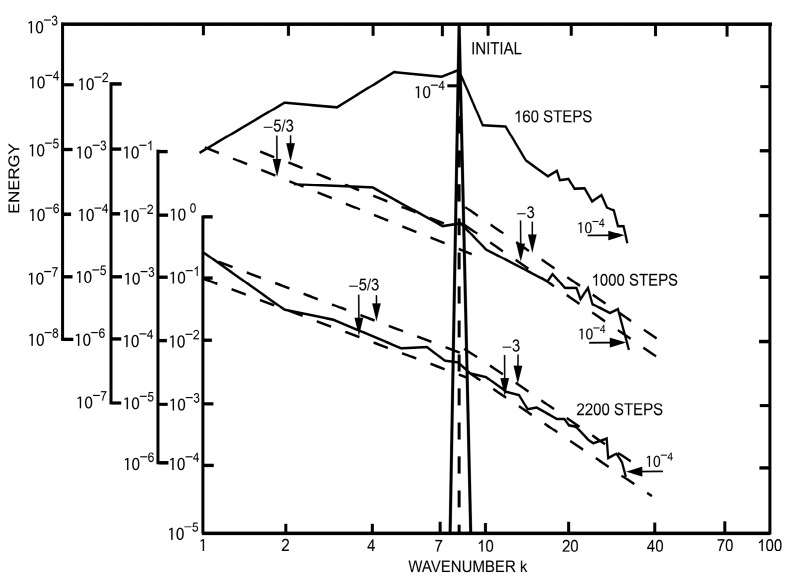
For comparisons of the power laws describing the spectra of the inertial energy and the enstrophy range early computer simulations of Lilly [86] have already been cited and interpreted by Kraichnan [70]. These numerical experiments show a convergence toward the predicted values of the power law exponents, which for the highest computation time (2200 time steps) are at low wave number close to −5/3 and at high wave number close to the equilibrium value −3. From Lilly [86] with changes.

With Kolmogorov’s assumption that the energy spectrum depends only on the wave number modulus *k* and the dissipation rate *ε*, the exponent −5/3 power law is obtained for two as well as for three dimensional turbulence (see [87] and Figure 5). Notice that this spectrum is valid only at low wave numbers up to an intersecting value $k_{I}$ after which a $k^{-3}$ power law (see also Figure 5) is observed that dominates the behaviour up to Kolmogorov’s dissipation wave number ([70,87]). 

Furthermore, notice also that a Kolmogorov-Oboukov turbulent flow is thermodynamically equally a non-equilibrium process that, on the other hand, shows simplicity by its lack of intermittency. For Re→∞, turbulent fields converge toward a state obeying the Kolmogorov-Oboukov energy spectrum. Tsallis [59] wrote that chaotic systems with strong chaos (showing at least one positive Lyapunov exponent) approach thermodynamic equilibrium.

Weakly chaotic and turbulent systems show complexer behavior than infinite Reynolds number turbulent flows. These systems are closer to criticality. Table 4, in its last line, shows that turbulent systems usually are characterized by *q* values between 5/3 and 3. Therefore, with (127) also the next refined energy spectrum follows:

(129) $$S(k)=\mathrm{const}k^{-\left(5/3+\mu \right)}.$$

The intermittency exponent *μ* is closely related to the structure function $\varsigma_{p}$ of order *p* of the fluid velocity (see [3]). From the *q*-value range in the last line of Table 4, we also deduce that:

(130) $$q=\frac{5}{3}+\mu ,\mu =\frac{d-D}{3},0\leq \mu \leq \frac{4}{3},$$

and conclude that Tsallis’ *q* factor copes directly with basic quantities of research on turbulence, which are known for many decades. Note that a power law exponent with a magnitude larger than 5/3 was a hypothesis of Kolmogorov [88] and Oboukov [89] for an intermittency correction (see also in [46]), which by the present analysis is more than just qualitatively confirmed. A comparison of Equation (130) with a corresponding formula in [22] reveals Equation (130). In this equation *d* denotes the spatial dimension and *D* the Hausdorff-Besicovitch dimension, which is a fractal dimension. The difference *d*-*D* is called codimension. All this is in order as long as one works with Lévy flight statistics (see e.g., [90,91,92]), where a cascade of Lévy flight sizes directly relates to corresponding diameters of eddies in a way that basic kinematic laws of turning eddies with different angular velocities are fulfilled (see [22]). This is also in full agreement with results of the fractal *β*-model [3]. On the other hand, Gotoh and Kraichnan [90] critically comment on the application of Tsallis’ non-equilibrium thermodynamics (with a constant *q*-factor) to turbulence. Arimitsu and Arimitsu [93] make the link of Tsallis thermodynamics to the multifractal model of turbulence. In this model the intermittency and, thereby, its exponent *μ* are scale dependent. Notice that intermittency increases with decreasing scale. These considerations are in perfect agreement with the direct relation (130) between *μ* and *q*, where a scale dependent intermittency exponent also calls for a scale-dependent *q* factor. In the language of Tsallis thermodynamics, different wave number sub systems show different *q* values.

*The second* is the large wave number regime, for which, from Equation (125), it follows that:

(131) $${\hat{\overset{⌣}{E}}}_{\varepsilon }(k)=\frac{1}{2\beta }\frac{1}{Z_{ES\varepsilon }}\frac{1}{q^{2}}\frac{1}{k^{2q}}.$$

Now, by applying Equation (119), the fractional derivative of this function is derived, which yields:

(132) $$S_{\varepsilon }(k)=const\frac{1}{k^{3q}},$$

which for *q* = 1 is identical with Kraichnan’s former result (56), derived with BG equilibrium thermodynamics, showing an ‘exponent minus three power law’. Also this result is in good agreement with the numerical experiments of Lilly [86], presented in Figure 5. The good coincidence between the equilibrium thermodynamic theory of Kraichnan, being the Tsallis thermodynamics special case with *q* = 1, and the experiments suggests that small eddies, which have smaller turnover and life times compared to large eddies (see Section 9) are close to or even exactly in thermal equilibrium. If in future experimentally determined small deviations to the power law exponent “−3” would be observed, then, with high probability they would be due to small nonequilibrium effects. In this context, note that Kraichnan proposed a small logarithmic correction to the enstrophy range of the spectrum [70].

## 9. Justification of the Quadratic Form of the Energy as a Functional of Real Space Coordinates

In this section we give a motivation of the quadratic form of the energy functional, which Kraichnan discovered. From the constancy of the real space and velocity variables (see below), viz.:

(133) $${\vec{\tilde{u}}}^{2}=const,\vec{\tilde{x}}=const\Rightarrow \vec{\tilde{x}}+{\vec{\tilde{u}}}^{2}=const,$$

also it follows the constancy of its sum (for the dimension of $\vec{\tilde{x}}$ see explanation following Equation (160)). This explains the statement of Kraichnan [70] that the constant energy surfaces in phase space are hyperspheres.

To describe the eddy fluctuation motion, we start with a *2-d* coupled dynamical system

(134) $${\dot{x}}_{1}=\omega \;x_{2},{\dot{x}}_{2}=-\omega \;x_{1},$$

where for simplicity we neglected all the wavy overbars. Differentiation of (Equation (134), left) leads to

(135) $${\ddot{x}}_{1}=\omega \;{\dot{x}}_{2}.$$

Substituting (Equation (134), right) into (135) then yields

(136) $${\ddot{x}}_{1}=-\omega \;{\dot{x}}_{1}.$$

By differentiating (Equation (134), right) and substituting (Equation (134), left) a second second-order differential equation follows, so that we now face a system of two ordinary differential equations

(137) $${\ddot{x}}_{1}=-\omega^{2}x_{2},{\ddot{x}}_{2}=-\omega^{2}\;x_{2}.$$

These are differential equations of harmonic oscillators for the two coordinates $x_{1}$ and $x_{2}$ with the solutions:

(138) $$x_{1}=A\sin(\omega \;t+\varphi ),x_{2}=A\cos(\omega \;t+\varphi ).$$

The squared velocity is:

(139) $${\vec{u}}^{2}=u_{1}^{2}+u_{2}^{2}={\dot{x}}_{1}^{2}+{\dot{x}}_{2}^{2}.$$

Substituting Equations (134), it follows that:

(140) $${\vec{u}}^{2}=\omega^{2}\left(x_{1}^{2}+x_{2}^{2}\right)=\left(\omega^{2}r^{2}\right)={\left(\omega \;r\right)}^{2}.$$

By a substitution of solution (138) into (139) and the application of Pythagora’s law for the trigonometric functions the same result is obtained. The energy of this rotating eddy is:

(141) $$E=\frac{1}{2}{\vec{u}}^{2}=\frac{1}{2}\omega^{2}r^{2}=const.$$

In a usual harmonic oscillator, the total energy *E* is the sum of the kinetic energy *T* and the potential energy *U*, which is constant. In the oscillation process these two exchange their energies under the restriction:

(142) E=T+U=const.

On the other hand, in a turbulent eddy there is an exchange between two kinetic energies of the two coupled oscillators:

(143) $$E=T_{1}+T_{2}=\frac{1}{2}u_{1}^{2}+\frac{1}{2}u_{2}^{2}=\frac{1}{2}{\vec{u}}^{2}=const.$$

We now rewrite this in the notation of the fractal beta model (see [3]) by renaming *r* by *l/2* and assigning the index 1 to several quantities:

(144) $$E_{1}=\frac{1}{2}{\vec{u}}_{1}{}^{2}=\frac{1}{8}\omega_{1}{}^{2}l_{1}^{2}=\frac{1}{8}\omega_{1}{}^{2}\left(x_{1}^{2}+x_{2}^{2}\right),r=\frac{l_{1}}{2}.$$

Notice that in turbulence research the largest eddy is denoted by the index 0. To cope with Kraichnan’s notation, we start the summations with index 1. Kraichnan stated that the total energy *E* is constant:

(145) $$E=\sum_{n=1}^{N}E_{n}=\mathrm{const}.$$

**Figure 6:**
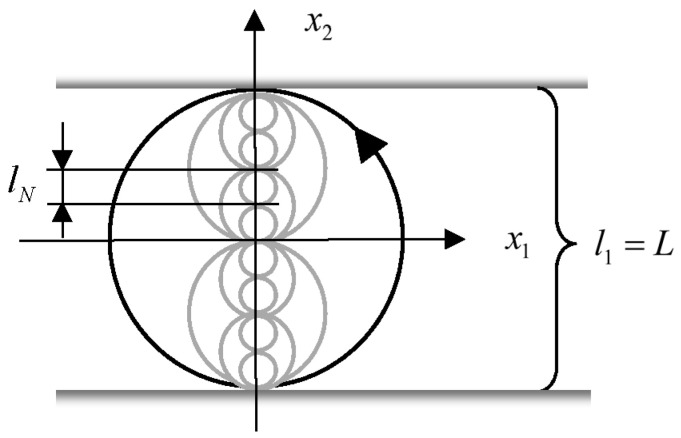
A schematic sketch of a hierarchy of *N* = 4 eddy classes. The largest eddies (an example is shown by the bold circle) show an extension, respectively diameter, $l_{1}$, which is identical to the width *L* of the fluid basin. Eddies of index *n* decay into a larger amount of smaller eddies characterized by index *n* + 1. Eddies of diameter $l_{N}≅l_{K}$ and smaller are destroyed by dissipation. The restrictive limitations toward large and small-scale eddies is taken into account by the Galerkin truncation method applied in the theory of this article.

Now, we take advantage of the fractal-*β* model (see [3]). Thereby, we follow the presentation in [22]. In this model eddies are assumed to occur in classes of different sizes $l_{n}$ (see Figure 6). The largest eddies show a diameter $l_{1}$. The ratio of eddy sizes of two neighboring classes is:

(146) $$\frac{l_{n+1}}{l_{n}}=\frac{1}{b},b>0,$$

where, following an assumption by Kolmogorov, in Figure 6 we have chosen a decay of eddies to such of half size (*b* = 2). Notice that *b* is an important parameter of a Lévy flight distribution, which in a special fractal model is connected with the eddy size ratio (see [22]). Now, it follows that:

(147) $$\frac{l_{n+1}}{l_{1}}=\frac{l_{n+1}}{l_{n}}\frac{l_{n}}{l_{n-1}}....\frac{l_{2}}{l_{1}}={\left(\frac{1}{b}\right)}^{n}={\left(\frac{1}{2}\right)}^{n}.$$

In the fractal *β*-model eddies do not fully occupy the available space. In each step from one eddy class to the next a reduction by the factor *β* (0 < *β* < 1) occurs, where this *β* gave the model its name. Then, the active space fraction is:

(148) $$p_{n}^{\left(active\right)}=\beta^{n-1}={\left(\frac{l_{n}}{l_{1}}\right)}^{d-D}.$$

Here, we do not need to specify the spatial dimension. However, in *2-d* turbulence one could imagine that the excessively large eddies are perfectly two dimensional, whereas finer structures (smaller eddies) fluctuate in all three spatial directions. It is assumed that at some intermediate wave number a small transition region occurs. A first assumption is that the eddies of the first class fully fill the available space. From Equation (148) it follows that:

(149) $$p_{1}^{\left(active\right)}=\beta^{0}=1.$$

Then an increasing intermittency to smaller scales is observed. The kinetic fluctuation energy in an eddy of class *n* is:

(150) $$E_{n}=\frac{1}{2}p_{n}^{\left(active\right)}\rho \;u_{n}^{2}=\frac{1}{2}\rho \;u_{n}^{2}{\left(\frac{l_{n}}{l_{1}}\right)}^{d-D}.$$

From this it follows that the velocity of eddies of class *n* is given by:

(151) $$u_{n}={\left(2\frac{E_{n}}{\rho }\right)}^{1/2}{\left(\frac{l_{n}}{l_{1}}\right)}^{\left(D-d\right)/2}.$$

The time in which the energy in an eddy of class *n* is transferred to one of class *n* + 1 is assumed to be the time of a single turn of an eddy of class *n*; consequently, this time is called ‘turnover time’ of an eddy:

(152) $$t_{n}=\frac{2\pi }{\omega_{n}}=\frac{l_{n}}{u_{n}}={\left(\frac{\rho }{2E_{n}}\right)}^{1/2}l_{1}^{(D-d)/2}l_{n}{}^{1+\left(d-D\right)/2}.$$

For further remarks on $t_{n}$ see Ref. [22]. The locality hypothesis of Kolmogorov [87] states that the transfer rate of energy from an eddy of one to its next class is constant and given by:

(153) $$\varepsilon_{n}=\frac{E_{n}}{t_{n}}=\varepsilon =const.$$

The range in which this is the case is called the inertial range. Now, it follows that:

(154) $$E_{n}=\varepsilon \;t_{n}=\varepsilon \;{\left(\frac{\rho }{2E_{n}}\right)}^{1/2}l_{1}^{(D-d)/2}l_{n}{}^{1+\left(d-D\right)/2},$$

where Equation (152) was substituted into (154). From this it follows that:

(155) $$E_{n}={\left(\frac{\rho }{2}\varepsilon^{2}\right)}^{1/3}l_{1}^{(D-d)/3}l_{n}{}^{2/3+\left(d-D\right)/3}.$$

For the largest eddies one has:

(156) $$E_{1}={\left(\frac{\rho }{2}\varepsilon^{2}\right)}^{1/3}l_{1}^{(D-d)/3}\;l_{1}{}^{2/3+\left(d-D\right)/3}\Rightarrow {\left(\frac{\rho }{2}\varepsilon^{2}\right)}^{1/3}=E_{1}\;l_{1}^{(d-D)/3}\;l_{1}{}^{-2/3+\left(D-d\right)/3},$$

where by writing in Equation (156) with intention twice $l_{1}$ with their specific exponents each, simplifies the following calculus. Now, by substituting Equation (156) into (155) yields:

(157) $$E_{n}=E_{1}\;{\left(\frac{l_{n}}{l_{1}}\right)}^{2/3+\left(d-D\right)/3}.$$

A comparison of this exponent with Equation (130) reveals that:

(158) $$\frac{2}{3}+\frac{d-D}{3}=q-1,$$

with the Tsallis parameter *q*. Now, Equation (157) is rewritten:

(159) $$E_{n}=E_{1}\;{\left(\frac{l_{n}}{l_{1}}\right)}^{q-1}.$$

With Equation (147) it follows from (159) that:

(160) $$E_{n}=E_{1}\;{\left[{\left(\frac{1}{b}\right)}^{n-1}\right]}^{q-1}=E_{1}\;{\left(b^{1-q}\right)}^{n-1}\Rightarrow \frac{E_{n+1}}{E_{n}}=b^{1-q}<1,b>1,q>1.$$

To obtain the total energy of the eddies of all classes, this equation is inserted into the sum of Equation (145):

(161) $$E=E_{1}\;\sum_{n=1}^{N}{\left(b^{1-q}\right)}^{n-1}=const.$$

The final result is:

(162) $$E=\frac{1-b^{N\left(1-q\right)}}{1-b^{1-q}}E_{1}=\kappa \;E_{1}.$$

This deserves the formal:

**Proposition** **4.** *By scaling laws the total turbulent kinetic energy can be expressed as a function of the energy contained in the eddies of the largest size* $E_{1}$ *only. The turbulent kinetic energy of the eddies of the remaining classes* $E_{2}\ldots E_{N}$ *is described by the multiplicative factor κ − 1 times* $E_{1}$. *This is a direct consequence of the self-similarity of the eddy cascade*.

The special case *N* = 1 leads to the correct solution of a total energy belonging to that of the single class containing only largest eddies:

(163) $$E=E_{1}.$$

The special case *q* = 1 yields a division “0/0”. Therefore, to Equation (162) the rule of ‘Bernoulli-de l’Hôpital’ is applied by differentiating the nominator and denominator with respect to *b*, yielding:

(164) $$E=Nb^{\left(N-1\right)\;\left(1-q\right)}E_{1},$$

which leads to the energy equipartition law between the eddies of different classes:

(165) $$E=NE_{1}.$$

For emphasis, the results (160) and (165) suggest:

**Proposition** **5.** *Equilibrium BG-turbulent flows show energy equipartition in the eddies of all classes and, consequently, for infinite Reynolds number flow its turbulent kinetic energy diverges*.

Next, with Equations (144) and (162) takes the form:

(166) $$E=\frac{1}{8}\kappa \;\omega_{1}{}^{2}\left(x_{1}^{2}+x_{2}^{2}\right).$$

By modifying the coordinates by the stretching transformation ${\overset{⌢}{x}}_{i}=\sqrt{\kappa /8}\;\omega_{1}x_{i},\;i\in \left\{1,2\right\}$—note that this space coordinate has the dimension of a velocity—one finds:

(167) $$u^{2}=\left({\overset{⌢}{x}}_{1}^{2}+{\overset{⌢}{x}}_{2}^{2}\right),$$

which is the result that also confirms Equation (A8) in the Appendix A below, q.e.d. 

The same results, with a different constant *κ*, could also be derived by taking in Equation (162) the occupation probability into account. This type of modeling includes the birth rates and life times of eddies of different classes (for details see [22]).

**Proposition** **6.** *By applying a Ritz-Galerkin truncation the continuous spectrum of turbulent flows is discretized. It belongs to a system of 2N coupled oscillators with N pairs each with its own fluctuation frequency. The large number of frequencies* $\omega_{n},n\in \left\{1,\ldots N\right\}$ *scale in a self-similar manner*.

A description by periodic or quasi-periodic motion finally rests on a somewhat crude model of turbulence. However, this does not shed any remarkable shadow on this kind of valuable description of isotropic turbulence. Rather, Kraichnan’s model and its generalization in this article offers considerable deep insights into the physics of *2-d* turbulent flows, as it will also become clear in the next section.

## 10. The Lévy Flight Probability Distribution and the Weakly *q*-Deformed Gaussian Distribution 

In Section 5 we were working with the Non-Gaussian Lévy flight probability distribution (103). On the other hand, in Section 8 the proof of Equation (125) was performed with a weakly *q*-deformed Gaussian probability distribution (see insert in Equation (A3) below). Therefore, the question could be raised, whether this might be an inconsistency in our modeling. However, in this section profound explanations are given on these two distributions, and it is shown that they are so closely related that in the present physical modeling no claim is justified that the procedure is deprived by rationality.

We start by clarifying what we define as a weakly *q*-deformed Gaussian distribution and what was already earlier defined to be a strongly or just a *q*-deformed Gaussian distribution.

*Firstly*, a weakly *q*-deformed Gaussian distribution is proposed by writing a usual Gaussian probability distribution as an escort probability distribution by combining Equation (97) with (105):

(168) $$P(x)=\frac{{\left[\exp\left(-\frac{x^{2}}{2\sigma^{2}}\right)\right]}^{q}}{Z_{ES_{\varepsilon }}},Z_{ES_{\varepsilon }}=\int_{0}^{\infty }{\left[\exp\left(-\frac{x^{2}}{2\sigma^{2}}\right)\right]}^{q}dx.$$

Then, it follows that:

(169) $$P(x)=\frac{\exp\left(-q\frac{x^{2}}{2\sigma^{2}}\right)}{Z_{ES_{\varepsilon }}}.$$

As demanded, the case *q* = 1 leads to the original classical Gaussian probability distribution (97). Definition (169) was applied in Equation (A4), and the motivation for this choice is given below.

*Secondly*, according to e.g., [94,95], a *q*-deformed Gaussian distribution is defined by applying Tsallis’ generalized exponential function (70):

(170) $$P(x)=\frac{e_{q}\left(\frac{x^{2}}{2\sigma_{q}{}^{2}}\right)}{Z_{q}}=\frac{1}{Z_{q}}{\left[1+\left(1-q\right)\frac{x^{2}}{2\sigma_{q}^{2}}\right]}^{1/\left(1-q\right)},\sigma_{1}=\sigma ,$$

where also here, based on considerations in Section 4, for *q* = 1 the classical Gaussian distribution (97) follows. The generalized standard deviation $\sigma_{q}$ is defined by Equation (101).

It is Kraichnan and his co-author Gotoh [90] who give us confirmation that the weakly deformed *q*-Gaussian probability distribution is the right choice in the context of our work. They state that with the Ritz-Galerkin description of the truncated dynamical system and the three constraints:

(a) *E* is a sum of energy contributions $E_{n}$: $E=\frac{1}{2}\sum_{n=1}^{N}E_{n}$ (see Equation (41)),
(b) The dynamics conserves energy, $E=\frac{1}{2}{\tilde{u}}^{2}=const$ (see Equation (143)),
(c) The Liouville property $\sum_{n=1}^{N}\frac{\partial {\dot{\tilde{x}}}_{n}}{\partial {\tilde{x}}_{n}}=0$ holds (see Equation (34)),

in phase space, an energy form exists that is a hyper sphere. Then also when *q* ≠ 1, the energy shell shows a homogenous probability density, and if additionally, *N* → ∞, the probability distribution converges toward the Gaussian distribution:

(171) $$P_{n}({\tilde{x}}_{n})=\frac{1}{\tilde{u}}\sqrt{\frac{N}{2\pi }}\exp\left(-\frac{1}{2}\frac{N\;{\tilde{x}}_{n}^{2}}{{\tilde{u}}^{2}}\right).$$

Note, that we assume that our finite dimensional dynamical system contains a very high number (2*N*) of binary oscillators, so that in equations following (171), also for finite *N*, we will write instead of the symbol “≈” the sign “=”. Furthermore, we know that in turbulence dissipation occurs, an effect which destroys the Liouville property c). On the other hand, in a delicate equilibrium between a random forcing and dissipation, which e.g., can be described by a (fractional) Langevin equation, Tsallis thermodynamics can be extended to weakly *q*-deformed Gaussian distributions as applied in our proof in the Appendix A, which is motivated by Gotoh and Kraichnan’s arguments above. 

Next, with the statement b) above, we generalize with help of (105) distribution (171) as:

(172) $$P(x)={\left(\frac{1}{u}\sqrt{\frac{N}{2\pi }}\right)}^{q}\exp\left(-\frac{q}{2}\frac{N\;x^{2}}{u^{2}}\right),$$

in which tildes have been dropped and where we have written $x^{2}\;\mathrm{for}\;{\left|\vec{x}\right|}^{2}$. Our approach is based on the escort generalized Boltzmann probability distribution and yields:

(173) $$P(x)=p^{q}(x)=\frac{1}{Z_{ES_{\varepsilon }}}{\left[\exp\left(-\beta_{q}\;E\right)\right]}^{q}=\frac{1}{Z_{ES_{\varepsilon }}}\exp\left(-q\beta_{q}\;E\right).$$

By inserting the energy (51) (see also Equation (A9) below), it follows that:

(174) $$P(x)=\frac{1}{Z_{ES_{\varepsilon }}}\exp\left[-q\beta_{q}\;\left(\alpha +\beta \;k^{2q}\right)x^{2}\right].$$

Comparing the prefactors of Equations (172) and (174) yields:

(175) $$Z_{ES_{\varepsilon }}={\left(u\sqrt{\frac{2\pi }{N}}\right)}^{q},$$

and by comparing the arguments of the exponential functions, with $u^{2}=2E$, yields:

(176) $$E=\frac{1}{4}\frac{N}{\beta_{q}}\frac{1}{\alpha +\beta \;k^{2q}},$$

which, with $Z_{ES\varepsilon }=2\beta_{q}/\left(q^{2}N\right)$, is consistent with Equation (125). Notice that the constant in Equation (176) depends on the space dilatation or contraction given in front of Equation (167)! Next, Equation (172) is rewritten as:

(177) $$P(x)={\left(\frac{1}{4\pi }\frac{N}{E}\right)}^{q/2}\exp\left(-\frac{1}{4}q\;\frac{1-q}{1-q}\frac{N}{E}x^{2}\right)={\left(\frac{1}{4\pi }\frac{N}{E}\right)}^{q/2}\exp{\left(-\frac{1}{4}q\left(1-q\right)\frac{N}{E}x^{2}\right)}^{1/\left(1-q\right)}.$$

Assuming that the argument in the exponential function in (177) is an order of magnitude smalller than unity, a linear Taylor series expansion is applied to yield:

(178) $$P(x)={\left(\frac{1}{4\pi }\frac{N}{E}\right)}^{q/2}{\left(1+\frac{1}{4}q\left(q-1\right)\frac{N}{E}x^{2}\right)}^{1/\left(1-q\right)},Z_{ES\varepsilon }={\left(4\pi \frac{E}{N}\right)}^{q/2}.$$

In the next step the prefactor on the left-hand side of (178) is incorporated into the squared brackets; this yields:

(179) $$P(x)={\left[{\left(\frac{1}{4\pi }\frac{N}{E}\right)}^{q\left(1-q\right)/2}+\frac{1}{4}q\left(q-1\right)\;{\left(\frac{1}{4\pi }\right)}^{q\left(1-q\right)/2}{\left(\frac{N}{E}\right)}^{q\left(1-q\right)/2+1}x^{2}\right]}^{1/\left(1-q\right)}.$$

The first term in the parenthesis is compared with the first one of Equation (103). This process yields:

(180) $$\lambda_{1}=\frac{kq}{q-1}{\left(\frac{1}{4\pi }\frac{N}{E}\right)}^{q\left(q-1\right)/2}\Rightarrow \lambda_{1}=\frac{kq}{q-1}Z_{ES\varepsilon }{}^{1-q}.$$

The same procedure for the second terms of Equations (103) and (179) and solving the emerging equation for $\lambda_{2}$ leads to:

(181) $$\lambda_{2}=\frac{1}{4}\left(q-1\right)\;{\left(\frac{1}{4\pi }\right)}^{q\left(1-q\right)/2}{\left(\frac{N}{E}\right)}^{q\left(1-q\right)/2+1}\lambda_{1}.$$

Inserting (180) results in:

(182) $$\lambda_{2}=\frac{1}{4}kq\;\frac{N}{E}.$$

Next, we consider the BG special case. Inserting *q* = 1 into Equation (180) delivers:

(183) $$\lambda_{1}=\underset{q\to 1}{\lim}\frac{k}{\left(q-1\right)}\to \infty ,$$

which corresponds to a diverging first Lagrange parameter. The limit *q* → 1 applied to Equation (182) reveals:

(184) $$\lambda_{2}=\frac{1}{4}k\;\frac{N}{E},$$

and when we apply Equation (165):

(185) $$\lambda_{2}=\frac{1}{4}\frac{k}{E_{1}}.$$

Next, we introduce a ‘generalized temperature’ of turbulent flows. We assume that eddies of class *n* with energy $E_{n}$ are characterized by their generalized temperature $T_{n}$. Thermodynamics suggests that the relation between energy and generalized temperature is:

(186) $$E_{n}=k\;T_{n},n\in \left\{1,2,\ldots ,\;N\right\}.$$

We may call $T_{n}$ the generalized turbulent Kelvin temperature of an eddy of class *n*. Applying this relation with *n* = 1 to Equation (185) yields:

(187) $$T_{1}=\frac{1}{4}\frac{1}{\lambda_{2}}.$$

This leads us to:

**Proposition** **7.** *In turbulent BG flows the inverse second Lagrange parameter* $\lambda_{2}$ *defines the generalized temperature of the eddies being members of the first class*.

Next, we apply the energy equipartition law of equilibrium turbulent flows (see Equation (160)), viz.:

(188) $$E_{1}=E_{2}=\ldots =E_{N-1}=E_{N}$$

and conclude with help of (160) and (186) for the entire energy-enstrophy spectrum that:

(189) $$T_{1}>T_{2}>\ldots >T_{I-1}>T_{I}=T_{I+1}\ldots =T_{N-1}=T_{N}.$$

By applying this new result to *2-d* turbulence, we now create:

**Proposition** **8.** *In 2-d turbulence eddies in the energy transfer range belonging to the classes n = 1, 2, …, I (I = intersection between the two ranges characterized by the wavenumber* $k_{I}$*) show decreasing generalized temperatures:* $T_{1}>T_{2}>\ldots >T_{I-1}>T_{I}$, *whereas the eddies in the enstrophy transfer range belonging to classes I + 1, I + 2, …; N − 1, N, show generalized temperatures that are equal:* $T_{I+1}=T_{I+2}=\ldots =T_{N-1}=T_{N}$. *This is a consequence that in the energy range the eddies are not in equilibrium, whereas in the enstrophy range they are*.

These findings are consistent with Kraichnan’s statement that in the enstrophy range, where eddies belonging to different wave number intervals are in thermal equilibrium, there is no energy transfer (see [69,70,86]).

Finally, it is stated that the probability distribution (103) and the escort generalized weak Gaussian probability distribution (172), applied in our model, do not lead to an inconsistency, because Equation (103) can be interpreted to be a first-order Taylor expansion of Equation (172). Equally satisfying is that comparisons between the two probability distributions lead to important correct statements on the equilibrium and non-equilibrium thermodynamics of eddies of different classes.

## 11. Discussion of Results, Conclusions and Outlook

This review is mainly based on work of Kraichnan [69,70] and Tsallis [59,64,78] and our extensions. A further important contribution, namely the relation between Tsallis thermodynamics and Lévy statistics is due to Alemany and Zanette [81]. Our paper incorporates and combines these works, but also provides a majority of new results; some remained unanswered and were hanging for decades as open ends. In this memoir a unique modern theory of *2-d* turbulence was created by e.g., implementing new ideas of fractional calculus, so that our new theory now presents itself with completeness and shows perfect agreement with experiments. We have also found a physically clearly defined generalized temperature of turbulent flows. Its variation acts as a measure of deviation of a turbulent system from equilibrium.

Kraichnan applied equilibrium BG thermodynamics to *2-d* and *3-d* turbulence. For *2-d* turbulence two partial regimes are observed, a low wave number regime, where energy is transferred down the cascade, and a large wave number regime in which the transported physical quantity is the enstrophy [96], flowing toward small wave numbers. The generalization of the *3-d* turbulence energy spectrum with Tsallis nonextensive formalism and fractional calculus seems promising, but has not yet been derived and is proposed for further investigation.

On the other hand, Tsallis has been a forerunner of generalizing thermodynamics to non-equilibrium processes. Turbulence at moderate Reynolds number is a highly non-equilibrium physical phenomenon and demands new nonlocal concepts that are still in their infancy. A large selection of different entropic forms was proposed, which luckily have some mutual connections, so that it is often more a matter of taste than correctness to apply one or another. The Tsallis entropic form works with a new formalism that generalizes the significant classical statistical based terms by a *q*-factor and, thus, leaves well-established formulas of equilibrium thermodynamics preserved and in a generalization to non-equilibrium thermodynamics practically unaltered, if the basic functions are *q*-generalized (e.g., by the *q*-exponential function, the *q*-logarithmic function or the *q*-deformed fractional derivative [54]). We introduced the weakly *q*-deformed Escort-Gaussian probability distribution, which is the right choice for a Ritz-Galerkin truncated system of the NSE that develops to a high-degree dynamical coupled system of 2*N* oscillators or *N* eddies, respectively. For a large number *N* of eddy classes, the Gaussian distribution is a good approximation and in the limit as *N* → ∞ it is even the correct one. We found that the Non-Gaussian Lévy probability distribution can be sought to be a Taylor expansion to this limiting case. However, it is well-known that most complex behavior occurs above criticality and at medium Reynolds numbers and simplifies in the infinite Reynolds number limit. 

According to a study of Alemany and Zanette (see [81]) Tsallis’ extended thermodynamics is directly related to Lévy walks and flights. And Lévy statistics had been applied to successfully describe turbulence (see e.g., [3,22])! A direct conclusion is that Tsallis’ contributions to statistical physics yield a favorite tool to build a solid foundation of thermodynamics of turbulence. However, Gotoh and Kraichnan [90] offered critical remarks concerning the application of Tsallis thermodynamics with a single *q*-value to turbulence. They also discussed the application of a wave number dependent *q* value to overcome occurring problems. Such emerge naturally in our article in the context of modeling turbulent fields with intermittency (compare with Equation (130)).

Furthermore, it is known that Lévy random walks and flights can be related to fractional Langevin and Fokker-Planck equations that are based on fractional calculus. It is evident that the dynamics belonging to Richardson’s and Mandelbrot’s fractal geometry is fractional dynamics and calculus. Egolf and Hutter [26] introduced fractional calculus to turbulence modeling. Therefore, it is a direct consequence that Tsallis’ thermodynamics can be formulated with the help of fractional calculus. Without this tool it is impossible to generate by a differentiation of a constant energy the Kolmogorov-Oboukov *k*^−5/3^ energy intensity spectrum. Moreover, a fractional generalization of the enstrophy is of importance to generalize Kraichnan’s energy and enstrophy intensity spectra.

With all these new developments, it was tempting for us to apply Tsallis’ thermodynamics to generalize Kraichnan’s important work on the Ritz-Galerkin truncated turbulent energy and enstrophy spectra of *2-d* turbulence. The new findings, related to the energy and enstrophy spectra, are consistent with three well-known types of descriptions of turbulence with an increasing level of complexity: (1) the BG thermodynamic results of Kraichnan; (2) the spectrum of Richardson, Oboukov and Kolmogorov; and (3) newer results of Lévy and Frisch, etc., describing turbulence including intermittency effects. 

In the context of the proof of the generalized *2-d* turbulence spectrum of Kraichnan, it has become clear that the usual definition of enstrophy with a square of the first derivative of the velocity relates intimately to Boltzmann-Gibbs (*q* = 1) equilibrium thermodynamics and requires a generalization with e.g., the nonextensive (*q* ≠ 1) Tsallis thermodynamics including fractional derivatives. In future, a generalization of the definition of enstrophy in articles and standard text books may be demanded.

All these new studies show that non-equilibrium thermodynamics and fractional calculus, involving nonlocality, are likely to play a crucial role in modern turbulence research and computation. Whereas such theoretical approaches have started to actively bloom, time seems now also ready for fluid dynamic computational experts to introduce into their numerical algorithms and codes new fractional concepts. Modern zero-equation turbulence models, as e.g., the DQTM [23], are more than just a compensation of the eliminated wave number terms [96] e.g., by a Ritz-Galerkin truncation method. They contain scaling laws and couple, in a statistical sense, small with large wave number eddies in a nonlocal manner. Therefore, we conjecture and predict that in future low-order fractional turbulence modeling will be superior and more accurate compared to the past and present results of higher-order conventional calculus computations, based on linear and local concepts, and will decrease the demanded computation power (CPU time) likely by at least an order of magnitude.

## Figures and Tables

**Table 1:** Physical systems with conventional linear (column 2) and with nonlinear behavior (column 3).

Problem	Linear	Nonlinear
Statistics	Gaussian	Non-Gaussian, e.g., Lévy distribution
Variance	Finite	Infinite
Scales	Single	Self-similar, power law
Diffusion	Brown	Anomalous
Thermodynamics	Equilibrium	Non-equilibrium
Entropy	Additive	Non-additive
Phase transition	Static	Dynamic

**Table 2:** The three main types of diffusivity (sub, normal and super diffusivity) are listed with a number of application examples.

Type of Diffusion	Examples
Subdiffusivity (dispersive diffusion)	Disordered media, fractal structures, trapping in condensed matter, sticking, glasses, etc.
Normal diffusivity (Brown’s diffusivity)	Monatomic gases, molecules, tracers, heat transfer, etc.
Superdiffusivity	Chaotic dynamics, phase diffusion in chaotic regimes of Josephson junctions, turbulence, etc.

**Table 3:** Characteristic values for the three diffusion domains. We remind you that α and *H* occur in Equation (92) and the succeeding text.

Diffusion	α	*h*	$\gamma_{L}$	*q*
sub	<1	<1/2	>2	−∞ < *q* < 5/3
normal	=1	=1/2	=2	=5/3
super	>1	>1/2	<2	5/3 < *q* ≤ 3

**Table 4:** Characteristic values of the three diffusion domains, some of their main contributors and the related thermodynamics.

*q*	Main Contributers	Thermodynamics
1	Kraichnan	Equilibrium
5/3	Kolmogorov-Oboukov	Non-equilibrium
>5/3	Lévy-Frisch	Non-equilibrium

## Appendix A. Proof of the Generalization of Kraichnan’s Energy-Enstrophy Spectrum

To determine the energy-enstrophy spectrum of *2-d* isotropic turbulence, we apply a Fourier transform to the generalized energy ${\overset{⌣}{E}}_{\varepsilon }(\vec{x})$:

(A1) $${\hat{\overset{⌣}{E}}}_{\varepsilon }(\vec{k})=\frac{1}{2\pi }\frac{1}{Z_{ES\varepsilon }}\int_{-\infty }^{\infty }{\overset{⌣}{E}}_{\varepsilon }\left(\vec{x}\right)P\left[{\overset{⌣}{E}}_{\varepsilon }\left(\vec{x}\right)\right]\;e^{-i\;\vec{k}\cdot \vec{x}}d\vec{x}.$$

This is identical to:

(A2) $${\hat{\overset{⌣}{E}}}_{\varepsilon }(\vec{k})=\frac{1}{2\pi }\frac{1}{Z_{ES\varepsilon }}\int_{-\infty }^{\infty }\int_{-\infty }^{\infty }{\overset{⌣}{E}}_{\varepsilon }\left(x_{1},x_{2}\right)P\left[{\overset{⌣}{E}}_{\varepsilon }\left(x_{1},x_{2}\right)\right]e^{-i\;\left(k_{1}x_{1}+k_{2}x_{2}\right)}dx_{1}dx_{2},$$

respectively:

(A3) $${\hat{\overset{⌣}{E}}}_{\varepsilon }(\vec{k})=\frac{1}{2\pi }\frac{1}{Z_{ES\varepsilon }}\int_{-\infty }^{\infty }\int_{-\infty }^{\infty }{\overset{⌣}{E}}_{\varepsilon }\left(x_{1},x_{2}\right)e^{-i\;\left(k_{1}x_{1}+k_{2}x_{2}\right)}{\left[e^{-\beta_{q}{\overset{⌣}{E}}_{\varepsilon }\left(x_{1},x_{2}\right)}\right]}^{q}dx_{1}dx_{2},$$

where the escort probability distribution function (105) was inserted. Then, this yields:

(A4) $${\hat{\overset{⌣}{E}}}_{\varepsilon }(\vec{k})=\frac{1}{2\pi }\frac{1}{Z_{ES\varepsilon }}\int_{-\infty }^{\infty }\int_{-\infty }^{\infty }{\overset{⌣}{E}}_{\varepsilon }\left(x_{1},x_{2}\right)e^{-i\;\left(k_{1}x_{1}+k_{2}x_{2}\right)}e^{-q\beta_{q}{\overset{⌣}{E}}_{\varepsilon }\left(x_{1},x_{2}\right)}dx_{1}dx_{2}.$$

It is clear that in the context of this proof also the enstrophy, Equation (42), requires a generalization by fractional calculus methods. Therefore, we introduce the modified enstrophy:

(A5) $${\tilde{\Omega }}_{\varepsilon }^{\left(q\right)}=\sum_{i,j=1}^{2}{\left|\frac{\partial^{q}{\tilde{u}}_{i}}{\partial x_{j}^{q}}\right|}^{2},\;D_{j}^{q}:=\frac{\partial^{q}}{\partial x_{j}^{q}},$$

with the fractional derivatives given by their two most common notations. Notice that, to perform this proof, we will not discuss which of the various fractional derivatives is best suited for this special application; this will be left for future clarification. The employed property of transformation of a fractional derivative of a certain order to a power law exponent with a value of exactly this order is valid for practically all versions of fractional derivatives, e.g., the Fourier fractional derivative [84], Riemann-Liouville fractional derivative [83], etc. It is straightforward to see that, with the help of Equations (41) and (48), this generalization leads to the following modification of Equation (51):

(A6) $${\tilde{E}}_{\varepsilon }^{\left(q\right)}=\left(\alpha +\beta \;k^{2q}\right){\hat{\tilde{u}}}^{2}=\left(\alpha +\beta \;k^{2q}\right)\sum_{k=n}^{N}{\hat{u}}_{n}^{2},$$

where a possibly occurring constant in front of $k^{2q}$ is absorbed in *β*.

Now, an important question is, whether in the fractal case the subspace conserving energy and enstrophy still remains a quadratic form. It is expected that in phase space this could be a fractal subset (strange attractor). However, in Section 9 we prove that even if the total energy presents itself in Euclidian form that it is based upon fractal features with self-similarity. Therefore, in agreement with Kraichnan we write:

(A7) $${\hat{\tilde{u}}}^{2}=a_{ik}{\tilde{x}}_{i}{\tilde{x}}_{k}=a_{11}{\tilde{x}}_{1}^{2}+a_{12}{\tilde{x}}_{1}{\tilde{x}}_{2}+a_{21}{\tilde{x}}_{2}{\tilde{x}}_{1}+a_{22}{\tilde{x}}_{2}^{2},a_{12}=a_{21}.$$

Then a rotational main axis transformation (${\tilde{x}}_{i}\to {\overset{⌢}{x}}_{i}$) is applied and the coordinates are compressed or stretched in such a manner that relation (A7) simplifies to:

(A8) $${\hat{\tilde{u}}}^{2}={\overset{⌢}{x}}_{1}^{2}+{\overset{⌢}{x}}_{2}^{2},$$

where in the following for simplicity the convex curved overheads will be dropped. In Section 9 we have already outlined a motivation and proof of this relation. Notice that this does not imply a decoupling of Fourier modes. It is evident that the sum of the replicas for each spatial component inserted into the square quantities of Equation (A8) describes self and interaction mode coupling. Now, it follows that:

(A9) $${\overset{⌣}{E}}_{\varepsilon }=\left(\alpha +\beta \;k^{2q}\right)\;\left(x_{1}^{2}+x_{2}^{2}\right).$$

This is inserted into (A4) and yields:

(A10) $$\begin{array}{ll} {\hat{\overset{⌣}{E}}}_{\varepsilon }(\vec{k})= & \frac{1}{2\pi }\frac{1}{Z_{ES\varepsilon }}\int_{-\infty }^{\infty }\int_{-\infty }^{\infty }\left(\alpha +\beta \;k^{2q}\right)\;\left(x_{1}^{2}+x_{2}^{2}\right)\;e^{-i\;\left(k_{1}x_{1}+k_{2}x_{2}\right)}e^{-q\beta_{q}\left(\alpha +\beta \;k^{2q}\right)\;\left(x_{1}^{2}+x_{2}^{2}\right)}dx_{1}dx_{2}=\\ & \frac{1}{2\pi }\frac{1}{Z_{ES\varepsilon }}\int_{-\infty }^{\infty }\int_{-\infty }^{\infty }\left(\alpha +\beta \;k^{2q}\right)\;x_{1}^{2}\;e^{-i\;\left(k_{1}x_{1}+k_{2}x_{2}\right)}e^{-q\beta_{q}\left(\alpha +\beta \;k^{2q}\right)\;\left(x_{1}^{2}+x_{2}^{2}\right)}dx_{1}dx_{2}+\\ & \frac{1}{2\pi }\frac{1}{Z_{ES\varepsilon }}\int_{-\infty }^{\infty }\int_{-\infty }^{\infty }\left(\alpha +\beta \;k^{2q}\right)\;x_{2}^{2}\;e^{-i\;\left(k_{1}x_{1}+k_{2}x_{2}\right)}e^{-q\beta_{q}\left(\alpha +\beta \;k^{2q}\right)\;\left(x_{1}^{2}+x_{2}^{2}\right)}dx_{1}dx_{2}=\\ & \frac{1}{2\pi }\frac{1}{Z_{ES\varepsilon }}\int_{-\infty }^{\infty }\int_{-\infty }^{\infty }\left(\alpha +\beta \;k^{2q}\right)\;x_{1}^{2}\;e^{-ik_{1}x_{1}\;}e^{-ik_{2}x_{2}\;}e^{-q\beta_{q}\left(\alpha +\beta \;k^{2q}\right)\;x_{1}^{2}\;}e^{-q\left(\alpha +\beta \;k^{2q}\right)\;x_{2}^{2}\;}dx_{1}dx_{2}+\\ & \frac{1}{2\pi }\frac{1}{Z_{ES\varepsilon }}\int_{-\infty }^{\infty }\int_{-\infty }^{\infty }\left(\alpha +\beta \;k^{2q}\right)\;x_{2}^{2}\;e^{-ik_{1}x_{1}\;}e^{-ik_{2}x_{2}\;}e^{-q\beta_{q}\left(\alpha +\beta \;k^{2q}\right)\;x_{1}^{2}\;}e^{-q\left(\alpha +\beta \;k^{2q}\right)\;x_{2}^{2}\;}dx_{1}dx_{2}. \end{array}$$

An advantage is that it is possible to separate the double-integrals in $x_{1}$ and $x_{2}$:

(A11) $$\begin{array}{ll} {\hat{\overset{⌣}{E}}}_{\varepsilon }(\vec{k})= & \frac{1}{Z_{ES\varepsilon }}\left[\frac{1}{\sqrt{2\pi }}\int_{-\infty }^{\infty }\left(\alpha +\beta \;k^{2q}\right)\;x_{1}^{2}\;e^{-ik_{1}x_{1}\;}e^{-q\beta_{q}\left(\alpha +\beta \;k^{2q}\right)\;x_{1}^{2}\;}dx_{1}\cdot \frac{1}{\sqrt{2\pi }}\int_{-\infty }^{\infty }e^{-ik_{2}x_{2}\;}e^{-q\left(\alpha +\beta \;k^{2q}\right)\;x_{2}^{2}\;}dx_{2}\right]+\\ & \frac{1}{Z_{ES\varepsilon }}\left[\frac{1}{\sqrt{2\pi }}\int_{-\infty }^{\infty }\left(\alpha +\beta \;k^{2q}\right)\;x_{2}^{2}\;e^{-ik_{2}x_{2}\;}e^{-q\beta_{q}\left(\alpha k^{2q}+\beta \right)\;x_{2}^{2}\;}dx_{2}\cdot \frac{1}{\sqrt{2\pi }}\int_{-\infty }^{\infty }e^{-ik_{1}x_{1}\;}e^{-q\left(\alpha +\beta \;k^{2q}\right)\;x_{1}^{2}\;}dx_{1}\right]. \end{array}$$

In this expression the various integrals are solved one after the other. The simpler two integrals are:

(A12) $$I_{i}^{\left(2\right)}=\frac{1}{\sqrt{2\pi }}\int_{-\infty }^{\infty }e^{-ik_{i}x_{i}\;}e^{-q\left(\alpha +\beta \;k^{2q}\right)\;x_{i}^{2}\;}dx_{i},i\in \left\{1,2\right\}.$$

With the abbreviation:

(A13) $$\gamma =2q\;\left(\alpha +\beta \;k^{2q}\right),$$

it follows that:

(A14) $$I_{i}^{\left(2\right)}=\frac{1}{\sqrt{2\pi }}\int_{-\infty }^{\infty }e^{-ik_{i}x_{i}-\frac{\gamma }{2}\;x_{i}^{2}\;}dx_{i},i\in \left\{1,2\right\}.$$

Furthermore, a quadratic expansion is performed:

(A15) $$I_{i}^{\left(2\right)}=\frac{1}{\sqrt{2\pi }}e^{-\frac{1}{2\gamma }\;k_{i}^{2}\;}\int_{-\infty }^{\infty }e^{-\frac{\gamma }{2}\;\left[x_{i}^{2}+\frac{2i}{\gamma }k_{i}x_{i}+{\left(\frac{ik_{i}}{\gamma }\right)}^{2}\right]\;}dx_{i},i\in \left\{1,2\right\},$$

which is identical to:

(A16) $$I_{i}^{\left(2\right)}=\frac{1}{\sqrt{2\pi }}e^{-\frac{1}{2\gamma }\;k_{i}^{2}\;}\int_{-\infty }^{\infty }e^{-\frac{\gamma }{2}\;{\left(x_{i}+\frac{ik_{i}}{\gamma }\right)}^{2}\;}dx_{i},i\in \left\{1,2\right\}.$$

A second abbreviation is introduced by the expression:

(A17) $$y_{i}=x_{i}+\frac{ik_{i}}{\gamma },\Rightarrow dy_{i}=dx_{i},i\in \left\{1,2\right\},$$

which simplifies Equation (A16) to:

(A18) $$I_{i}^{\left(2\right)}=\frac{1}{\sqrt{2\pi }}e^{-\frac{1}{2\gamma }\;k_{i}^{2}\;}\int_{-\infty }^{\infty }e^{-\frac{\gamma }{2}y_{i}^{2}}dy_{i},i\in \left\{1,2\right\}.$$

In Ref. [85] the following formula is listed for *a* > 0:

(A19) $$\int_{0}^{\infty }e^{-a^{2}x^{2}}dx=\sqrt{\frac{\pi }{2a}}\Rightarrow \int_{-\infty }^{\infty }e^{-\frac{\gamma }{2}y_{i}^{2}}dy_{i}=\sqrt{\frac{2\pi }{\gamma }},$$

which is directly inserted into (A18). This yields the final result for this integration problem:

(A20) $$I_{i}^{\left(2\right)}=\frac{1}{\gamma^{1/2}}e^{-\frac{1}{2\gamma }\;k_{i}^{2}},i\in \left\{1,2\right\}.$$

The two slightly larger integrals are:

(A21) $$I_{i}^{\left(1\right)}=\frac{1}{\sqrt{2\pi }}\int_{-\infty }^{\infty }\left(\alpha +\beta \;k^{2q}\right)\;x_{i}^{2}\;e^{-ik_{i}x_{i}\;}e^{-q\left(\alpha +\beta \;k^{2q}\right)\;x_{i}^{2}\;}dx_{i},i\in \left\{1,2\right\}.$$

With abbreviation (A13) this transforms to:

(A22) $$\begin{array}{l} I_{i}^{\left(1\right)}=\frac{1}{2\sqrt{2\pi }}\frac{\gamma }{q}\int_{-\infty }^{\infty }\;x_{i}^{2}\;e^{-ik_{i}x_{i}-\frac{\gamma }{2}\;x_{i}^{2}}\;dx_{i},i\in \left\{1,2\right\}. \end{array}$$

and with (A17) the result is:

(A23) $$I_{i}^{\left(1\right)}=\frac{1}{2\sqrt{2\pi }}\frac{\gamma }{q}e^{-\frac{1}{2\gamma }k_{i}^{2}}\int_{-\infty }^{\infty }\;{\left(y_{i}-\frac{ik_{i}}{\gamma }\right)}^{2}e^{-\frac{\gamma }{2}y_{i}^{2}}\;dy_{i},i\in \left\{1,2\right\}.$$

By working on the parenthesis this integral is separated into three integrals:

(A24) $$I_{i}^{\left(1\right)}=\frac{1}{2\sqrt{2\pi }}\frac{\gamma }{q}e^{-\frac{1}{2\gamma }k_{i}^{2}}\left(\int_{-\infty }^{\infty }\;y_{i}^{2}e^{-\frac{\gamma }{2}y_{i}^{2}}\;dy_{i}-2i\frac{k_{i}}{\gamma }\;\int_{-\infty }^{\infty }\;y_{i}\;e^{-\frac{\gamma }{2}y_{i}^{2}}\;dy_{i}-\frac{k_{i}^{2}}{\gamma^{2}\;}\int_{-\infty }^{\infty }\;e^{-\frac{\gamma }{2}y_{i}^{2}}\;dy_{i}\right).$$

The second integral, because of its antisymmetric nature, is zero; so Equation (A24) simplifies to:

(A25) $$I_{i}^{\left(1\right)}=\frac{1}{2\sqrt{2\pi }}\frac{\gamma }{q}e^{-\frac{1}{2\gamma }k_{i}^{2}}\int_{-\infty }^{\infty }\;y_{i}^{2}e^{-\frac{\gamma }{2}y_{i}^{2}}\;dy_{i}-\frac{1}{2\sqrt{2\pi }}\frac{k_{i}^{2}}{q\gamma }e^{-\frac{1}{2\gamma }k_{i}^{2}}\int_{-\infty }^{\infty }\;e^{-\frac{\gamma }{2}y_{i}^{2}}\;dy_{i}.$$

In Ref. [85] the following result can be found:

(A26) $$\int_{0}^{\infty }x^{2}e^{-a^{2}x^{2}}dx=\frac{\sqrt{\pi }}{4a^{3}}\Rightarrow \int_{-\infty }^{\infty }y_{i}^{2}e^{-\frac{\gamma }{2}y_{i}^{2}}dy_{i}=\sqrt{2\pi }\frac{1}{\gamma^{3/2}}.$$

With (A19) and (A26) it is concluded that:

(A27) $$I_{i}^{\left(1\right)}=\frac{1}{2q}\left(\frac{1}{\gamma^{1/2}}-\frac{k_{i}^{2}}{\gamma^{3/2}}\right)\;e^{-\frac{1}{2\gamma }k_{i}^{2}}.$$

Equation (A11) can be written as:

(A28) $${\hat{\overset{⌣}{E}}}_{\varepsilon }(\vec{k})=\frac{1}{Z_{ES\varepsilon }}\left(I_{1}^{\left(1\right)}\cdot I_{2}^{\left(2\right)}+I_{2}^{\left(1\right)}\cdot I_{1}^{\left(2\right)}\right)$$

Substituting the partial solutions of the integrals (A20) and (A27) into (A28), the fully outlined result is:

(A29) $${\hat{\overset{⌣}{E}}}_{\varepsilon }(\vec{k})=\frac{1}{Z_{ES\varepsilon }}\left[\frac{1}{2q}\left(\frac{1}{\gamma^{1/2}}-\frac{k_{1}^{2}}{\gamma^{3/2}}\right)\;e^{-\frac{1}{2\gamma }k_{1}^{2}}\cdot \frac{1}{\gamma^{1/2}}e^{-\frac{1}{2\gamma }\;k_{2}^{2}}+\frac{1}{2q}\left(\frac{1}{\gamma^{1/2}}-\frac{k_{2}^{2}}{\gamma^{3/2}}\right)\;e^{-\frac{1}{2\gamma }k_{2}^{2}}\cdot \frac{1}{\gamma^{1/2}}e^{-\frac{1}{2\gamma }\;k_{2}^{2}}\right],$$

which simplifies to:

(A30) $${\hat{\overset{⌣}{E}}}_{\varepsilon }(\vec{k})=\frac{1}{Z_{ES\varepsilon }}\frac{1}{q}\left[\left(\frac{1}{\gamma }-\frac{1}{2}\frac{k^{2}}{\gamma^{2}}\right)\right]\;e^{-\frac{1}{2\gamma }k^{2}},k=\sqrt{k_{1}^{2}+k_{2}^{2}}.$$

For *α* ≪ *β* and *k* ≪ ${\left(2\sqrt{\beta q}\right)}^{1/\left(1-q\right)}$, respective *β* ≪ *α* and *k* ≪ $2\sqrt{\alpha q}$, the second term in the squared brackets is neglected. This leads to the following expression:

(A31) $${\hat{\overset{⌣}{E}}}_{\varepsilon }(\vec{k})=\frac{1}{Z_{ES\varepsilon }}\frac{1}{q\gamma }e^{-\frac{1}{2\gamma }k^{2}}.$$

With a Taylor series expansion of the exponential function, respecting only the leading term, by applying the same argument again, one concludes that:

(A32) $${\hat{\overset{⌣}{E}}}_{\varepsilon }(\vec{k})=\frac{1}{Z_{ES\varepsilon }}\frac{1}{q\gamma }.$$

Substituting Equation (A13) into this equation leads to:

(A33) $${\hat{\overset{⌣}{E}}}_{\varepsilon }(\vec{k})=\frac{1}{2}\frac{1}{Z_{ES\varepsilon }}\frac{1}{q^{2}}\frac{1}{\alpha +\beta \;k^{2q}},$$

which is the proof for the intensity spectrum (125). For $q=\beta_{q}=Z_{BG\varepsilon }=1$ from (A33) it follows:

(A34) $${\hat{\overset{⌣}{E}}}_{\varepsilon }(\vec{k})=\frac{1}{2}\frac{1}{\alpha +\beta \;k^{2}},$$

called the Kraichnanian energy-enstrophy intensity spectrum (52), q.e.d.
